# Spatial vision in insects is facilitated by shaping the dynamics of visual input through behavioral action

**DOI:** 10.3389/fncir.2012.00108

**Published:** 2012-12-20

**Authors:** Martin Egelhaaf, Norbert Boeddeker, Roland Kern, Rafael Kurtz, Jens P. Lindemann

**Affiliations:** Neurobiology and Centre of Excellence “Cognitive Interaction Technology”Bielefeld University, Germany

**Keywords:** spatial behavior, optic flow, saccades, flying insects, obstacle avoidance, navigation behavior

## Abstract

Insects such as flies or bees, with their miniature brains, are able to control highly aerobatic flight maneuvres and to solve spatial vision tasks, such as avoiding collisions with obstacles, landing on objects, or even localizing a previously learnt inconspicuous goal on the basis of environmental cues. With regard to solving such spatial tasks, these insects still outperform man-made autonomous flying systems. To accomplish their extraordinary performance, flies and bees have been shown by their characteristic behavioral actions to actively shape the dynamics of the image flow on their eyes (“optic flow”). The neural processing of information about the spatial layout of the environment is greatly facilitated by segregating the rotational from the translational optic flow component through a saccadic flight and gaze strategy. This active vision strategy thus enables the nervous system to solve apparently complex spatial vision tasks in a particularly efficient and parsimonious way. The key idea of this review is that biological agents, such as flies or bees, acquire at least part of their strength as autonomous systems through active interactions with their environment and not by simply processing passively gained information about the world. These agent-environment interactions lead to adaptive behavior in surroundings of a wide range of complexity. Animals with even tiny brains, such as insects, are capable of performing extraordinarily well in their behavioral contexts by making optimal use of the closed action–perception loop. Model simulations and robotic implementations show that the smart biological mechanisms of motion computation and visually-guided flight control might be helpful to find technical solutions, for example, when designing micro air vehicles carrying a miniaturized, low-weight on-board processor.

## Optic flow as an important spatial cue for fast moving animals

Behavior is a phenomenon that takes place in space and is intricately entangled with it. The organism is required to interact with its surroundings in a way appropriate to the respective situational context. It should be able to respond appropriately to objects, for instance, by avoiding collisions with obstacles or by detecting and fixating inanimate objects of interest or other organisms, such as a predator, prey, or mate. On a larger spatial scale, organisms should be able to navigate from one place to another and to localize a goal on the basis of environmental spatial cues.

Insects are obviously well able to cope with these behavioral challenges in a highly virtuosic and efficient way. Think of a blowfly, for example, landing on the rim of a cup, or two flies chasing each other; without technical assistance, our visual system is incapable of resolving the complexity of such flight maneuvres, and the speed at which they are executed exceeds by far the capacities of our own motor system. During their virtuosic flight maneuvres, blowflies can make up to ten sudden (“saccadic”) turns per second, during which they may reach angular velocities of up to 4000°/s. The extraordinary navigational skills of bees are another awe inspiring example of insect spatial behavior: spatial cues enable bees to localize previously learnt, barely visible goals, such as a food source or the entrance to their nest, over large distances even in cluttered environments. All these feats are accomplished with visual systems of comparatively poor spatial resolution and extremely small brains that consist of no more than a million neurons, underlining the resource efficiency of the underlying mechanisms.

We will argue in this review that biological agents, such as flying insects, are such efficient and adaptive autonomous systems because they rely, to a large extent, on strategies by which they shape their sensory input through the specific way they move and change their gaze direction. In this way, they actively reduce the complexity of their sensory input and, thus, the computational load for the underlying brain mechanisms. Therefore, by exploiting the consequences of the action–perception cycle, animals with even tiny brains, such as insects, are enabled to perform extraordinarily well in solving spatial vision tasks in a wide range of behavioral contexts. This view somehow contrasts with common conceptions of how spatial vision is accomplished.

If laypeople are asked for the requirements of spatial vision, they are likely to reply that most animals, including humans, are equipped with two eyes which allow them to view the world from slightly different vantage points, and that the nervous system makes use of the resulting disparity information for depth vision. However, the spatial range that can be resolved in this way is critically restricted by the distance between the eyes, the overlap of their visual fields and their spatial resolution (Collett and Harkness, 1982). Hence, stereoscopic vision—if it is available at all to a particular animal species—is functional only in the near range. This poses a problem, especially for fast moving animals, such as many flying insects (as well as for human car drivers), because, in order to control appropriate reactions, such as avoiding collisions with obstacles, spatial information is required at much greater distances than may be available through stereoscopic mechanisms. Amongst the depth cues that are available in addition to binocular information, for example, contrast differences between near and distant objects (Collett and Harkness, 1982), the retinal image motion induced by self-movements of the animal (“optic flow”) is particularly relevant (Koenderink, 1986; Rogers, 1993; Poteser and Kral, 1995; Lappe, 2000; Redlick et al., 2001; Vaina et al., 2004).

**Figure 1:**
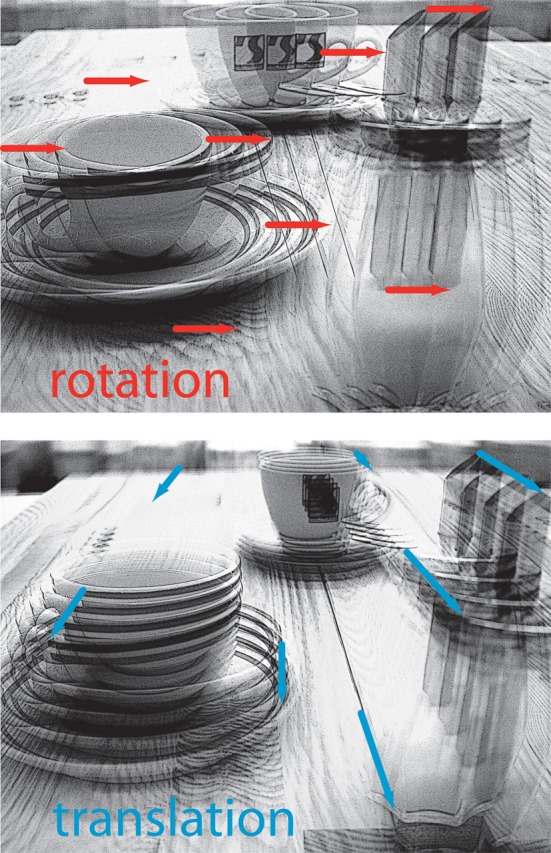
**Schematic illustration of the consequences of rotational (upper diagram) or translational self-motion (bottom diagram) for the resulting optic flow.** Superimposed images were either generated by rotating a camera around its vertical axis or by translating it forward. Rotational self-motion leads to image movements (red arrows) of the same velocity (reflected in the arrow length) irrespective of the distance of environmental objects from the observer. In contrast, the optic flow elicited by translational self-motion (blue arrows) depends on the distance between objects from the observer. Hence, translational optic flow contains spatial information.

Whenever an animal moves in its environment, the retinal images are continually displaced. During translatory movements, these displacements depend on the distance of environmental objects to the eyes, their angular location relative to the direction of motion and the velocity of locomotion. Only translational optic flow is distance dependent and, thus, contains spatial information, whereas rotational optic flow is useless for spatial vision, because all objects during rotations are displaced at the same angular velocity irrespective of their distance (Figure 1; Koenderink, 1986). Hence, the translatory optic flow component contains information about the relative distance of environmental objects from the animal: objects nearby pass quickly, while objects far-off appear virtually stationary. This motion-induced spatial information is based on behavioral action, because it is only available during self-motion, but not when the animal is stationary. Many animals, ranging from insects to humans, were concluded to exploit optic flow information for depth cueing.

We will focus in this review on the spatial behavior of insects that is based on depth information derived from optic flow. Since optic flow is particularly relevant during fast locomotion in three dimensions, we will mainly cover spatial vision in flight and address four major issues: (1) Components of insect behavior that are thought to be involved in solving basic spatial tasks and how they may depend on motion-based information; (2) the processing of motion-dependent spatial information and how it is facilitated by active gaze movements; (3) the representation of behaviorally relevant spatial information in the visual system; and (4) the behavioral significance of neurons extracting information about self-motion of the animal, as well as the environment, from the image flow generated on the eyes as a consequence of the action–perception loop being closed. Obviously, solving any spatial vision task—especially by flying insects that lack passive stability—requires, as a precondition, the animal's flight attitude to be somehow stabilized by appropriate feedback control systems. This issue, though very important for spatial orientation behavior and widely analysed for decades, will be touched on only briefly, because it has already been thoroughly reviewed (Hengstenberg, 1993; Taylor and Krapp, 2008).

## Behavior involved in spatial tasks and its control by visual motion cues

Many animals, including humans, use optic flow for the control of spatial behavior. Since spatial information can most easily be extracted from the retinal image flow during translatory self-motion, some animals execute translatory movements of their body and/or head that appear to be dedicated to generate optic flow suitable for depth cueing. Locusts, mantids, and dragonflies, for instance, sitting in ambush perform lateral body and head movements in preparation for a jump or for catching prey, respectively (Collett, 1978; Sobel, 1990; Collett and Paterson, 1991; Kral and Poteser, 1997; Olberg et al., 2005). Some bird species bob their heads back and forth, most likely to acquire depth information (Davies and Green, 1988; Necker, 2007). Moreover, flying insects, such as flies and bees (Schilstra and van Hateren, 1999; Boeddeker et al., 2010; Braun et al., 2010, 2012; Geurten et al., 2010), but also birds (Eckmeier et al., 2008), perform a saccadic flight and gaze strategy in which short and rapid head and body saccades are separated by largely translatory locomotion. This strategy facilitates access to spatial information from the resulting optic flow.

**Figure 2:**
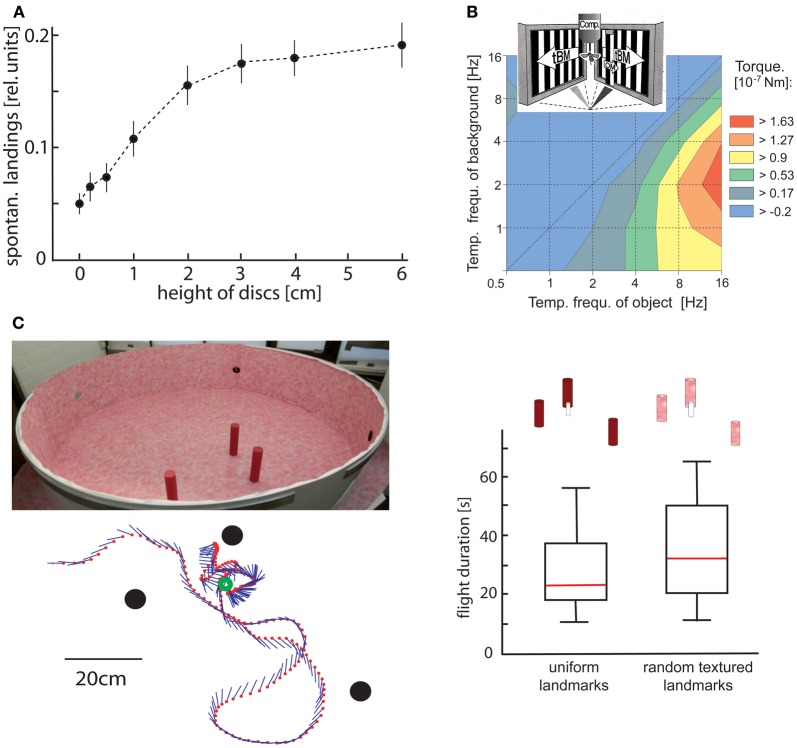
**Object detection by relative motion cues. (A)** Relative number of spontaneous landings of free-flying flies on discs covered with a random texture of different heights. The floor and walls of the flight arena were covered with the same texture. Hence, the discs could only be discriminated by relative motion cues induced on the eyes by the self-motion of the animal. Flies landed on discs raised at least 1 cm above the floor significantly more often than on a reference disc on the floor (data from Kimmerle et al., 1996). **(B)** Contour plot of the turning responses of tethered flying flies measured with a yaw torque compensator (comp) for different combinations of temporal frequencies of object motion (OM) and translatory background motion (tBM). The motion stimuli were striped patterns (spatial period 6.3°) presented on two monitor screens placed at an angle of 90° symmetrically in front of the fly. OM was displayed within a vertical 6.3° wide window in front of the right eye. Object-induced responses are given in a color coded way with warmer colors indicating larger responses. Flies show strong turning responses when OM is faster than tBM. The strongest responses are induced when the background is not stationary, but moves slowly (data from Kimmerle et al., 1997). **(C)** Landmark navigation of honeybees in a cylindrical flight arena with three cylindrical landmarks (upper left diagram). The landmarks were either homogeneously red or were covered by the same random pattern as the background. Bees were trained to find a barely visible feeder placed between the homogeneous landmarks. The trajectory of one search flight maneuvre is shown in the top view (bottom left diagram). The feeder (green circle) and the landmarks (black dots) are indicated. The position of the bee is indicated by red dots at each 32 ms interval; straight lines represent the orientation of the long axis of the bee. The duration of search flights until landing on the feeder was not significantly increased when the pattern of the landmarks was changed from homogeneous red to the random dot texture that also covered the background (right diagram). Red lines indicate median values, the upper and lower margins of the boxes, the 75th and 25th percentiles; the whiskers indicate the data range (Data from Dittmar et al., 2010).

The use of optic flow to gain spatial information has been shown most convincingly in behavioral experiments in which animals responded to objects that were camouflaged by covering them with the same texture as their background. Thus, these objects could be discriminated only on the basis of optic flow cues elicited during self-motion. *Drosophila*, for instance, is well able to discriminate the distance of different objects on the basis of slight differences in their retinal velocities (Schuster et al., 2002). Bees (Srinivasan et al., 1987; Lehrer et al., 1988) and blowflies (Kimmerle et al., 1996) use relative motion cues mainly at the edges of objects to discriminate between their height and to land on them (Figure 2A; Srinivasan et al., 1990; Kimmerle et al., 1996; Kern et al., 1997). Bees also use motion contrast in discrimination tasks (Lehrer and Campan, 2005) and for navigating back to the previously learnt location of a barely visible goal (Figure 2C; see below; Dittmar et al., 2010). Moreover, hawk-moths hovering in front of a flower use motion cues to control their distance to the nectar donating blossom (Pfaff and Varjú, 1991; Farina et al., 1994; Kern and Varjú, 1998). However, motion information is also used for spatial tasks that are not related to objects. Bees, for instance, exploit optic flow information to estimate distances traveled during navigation flights. The dependence of optic flow information on the depth structure of the environment is also relevant in this context: experimental manipulation of the environment between flights can induce characteristic errors in distance estimation because estimates of distances traveled in a given environment cannot be generalized to environments with different depth structures (Srinivasan et al., 2000; Esch et al., 2001; review: Wolf, 2011).

What are the mechanisms involved in solving spatial behavioral tasks? Insects play a pivotal role in systems analyses of these mechanisms, both at the behavioral and the neural level. Behavioral systems analyses have been mainly performed in flight simulators on tethered flying flies, because the visual input can be perfectly controlled by the experimenter while, in most experimental paradigms, turning responses are recorded. Here, the visual consequences of locomotion are emulated by motion stimuli to which the tethered animal is exposed. However, the degrees of freedom of movement that can be executed by the animal and monitored by the experimenter in these behavioral paradigms are constrained, thus providing only limited access to the rich behavioral repertoire of the animal. Apart from a few exceptions (e.g., Land and Collett, 1974; Collett and Land, 1975; Wagner, 1982; Zeil, 1986), it has only recently become possible to investigate spatial behavior systematically under free-flight conditions with high spatial and temporal resolution and to also reconstruct what an animal has seen during largely unconstrained behavior (Lindemann et al., 2003). In the following, we restrict the review to only a few components of spatial behavior that have been experimentally investigated in detail.

### Object detection and object-directed responses

It has been known for a long time from experiments in tethered flight that flies can discriminate objects from their background on the basis of motion cues and attempt to fixate them in the frontal visual field (Virsik and Reichardt, 1976; Reichardt and Poggio, 1979; Reichardt et al., 1983; Egelhaaf, 1985a; Egelhaaf and Borst, 1993a; Kimmerle et al., 1997, 2000; Maimon et al., 2008; Aptekar et al., 2012). In these experiments, the tethered animal could not move, and only its yaw torque was measured. Relative motion was generated by specifically controlling object and background displacements. In real life, this situation usually occurs as a consequence of the action–perception cycle being closed while the animal moves in a three-dimensional environment and actively generates relative motion cues on its eyes through its behavior (see above).

Only three features of the control system mediating object detection in flies will be mentioned here. (1) The detectability of objects depends to a large extent on the dynamical properties of object and background motion. Object detection is facilitated if the background moves at a moderate velocity, such as during translation in an environment where the background is at a medium distance from the animal (Figure 2B) (Kimmerle et al., 1997). (2) The visual pathways extracting motion-dependent object information and those processing other types of motion information (e.g., those controlling compensatory optomotor responses or translation velocity) are commonly assumed to segregate at the level of the fly's third visual neuropile. The object system appears to be distinguished by its dynamical and other properties. In particular, the object system responds to high-frequency changes of the retinal position and velocity of the object, whereas strong compensatory optomotor responses are evoked by low-frequency velocity changes (Egelhaaf, 1987; Aptekar et al., 2012). The object pathway appears to be kept separate from the other pathways up to the level of the steering muscles that mediate object-induced turns (Egelhaaf, 1989). (3) Even when the object moves exactly in the same way in subsequent stimulus presentations, it may either be fixated by the fly or no fixation responses may be elicited at all. Such a bimodal distribution of responses in the behavioral context of object detection—a full response or no response—suggests a gating mechanism in the neural pathway mediating motion-induced object fixation (Kimmerle et al., 2000).

Currently we can only speculate about the functional significance under real-life conditions of a control system that induces turning responses in tethered flight toward an object moving in front of its background. Potentially, an object may initiate landing behavior under free-flight conditions. This is plausible in blowflies as well as in bees, because (1) an object is most effective in eliciting fixation responses when the ventral part of the visual field is stimulated (Virsik and Reichardt, 1976), and (2) when detecting and approaching a landing site in free-flight, relative motion cues are exploited mainly in the ventral visual field (Wagner, 1982; Lehrer et al., 1988; Kimmerle et al., 1996; Kern et al., 1997; van Breugel and Dickinson, 2012). Similar object-detection systems could play an important role in bees during local navigation when landmarks based on contrast, texture, and relative motion cues need to be detected to guide the animal to its goal (see below).

### Collision avoidance

In many situations, objects or other structures in the environment (e.g., extended surfaces, such as walls) are not goals the animal may aim for, but may interfere with the animal's trajectory as obstacles that need to be avoided. Thus, collision avoidance represents a basic, but highly relevant spatial task. Again, optic flow has been shown in a variety of animals, including humans, to be one of the most relevant cues that may signal an impending collision (e.g., Lappe, 2000; Vaina et al., 2004).

Optic flow has been shown to be relevant in collision avoidance behavior for both tethered and free-flying flies. There is consensus amongst studies that asymmetries in the optic flow across the two eyes, for instance, when approaching environmental structures on one side, are decisive for eliciting collision avoidance responses: (1) Flies tend to turn away from the eye experiencing image expansion (Tammero and Dickinson, 2002a,b; Tammero et al., 2004; Bender and Dickinson, 2006b; Budick et al., 2007; Reiser and Dickinson, 2010). (2) The probability of eliciting an evasive turn has been concluded to be highest if the focus of image expansion is located in the lateral rather than in the frontal part of the visual field (Tammero and Dickinson, 2002a; Tammero et al., 2004; Bender and Dickinson, 2006b). Such optic flow might occur during flights with a strong sideways component. These results do not imply that the focus of expansion in the retinal motion pattern during object approach is explicitly extracted by the neuronal circuits that mediate collision avoidance. Based on experiments done in free-flight in different types of flight arenas that allow for more complex behavior than in tethered flight, mechanisms that rely on asymmetries in the optic flow field across the two eyes other than explicitly extracting the focus of expansion are well able to account for relevant aspects of collision avoidance (see below; Lindemann et al., 2008; Mronz and Lehmann, 2008; Kern et al., 2012).

### Interaction between object fixation and collision avoidance

Expanding visual flow fields are encountered by flying insects not only when they encounter an obstacle, but also when flying straight toward an object that may serve as a landing site or as a landmark in the context of navigation behavior. As sketched above, tethered flying *Drosophilae* turn away from an expanding retinal image. Given the strength of this evasive response, it is difficult to explain how flies can fly straight in natural surroundings with ample objects surrounding them. This apparent paradox is partially resolved by the finding that *Drosophila*, when flying toward a conspicuous object, tolerates a level of expansion that would otherwise induce avoidance (Reiser and Dickinson, 2010). This suggests that the gain of the control system mediating evasive turns is reduced if prominent visual features are attractive and represent a behavioral goal. Therefore, flies appear to require a goal to keep an overall flight direction, either toward a salient object (Heisenberg and Wolf, 1979; Götz, 1987; Maimon et al., 2008; Reiser and Dickinson, 2010), toward an attractive odorant (Budick and Dickinson, 2006), when flying upwind (Budick et al., 2007), or while pursuing a moving target such as a potential mate (Trischler et al., 2010).

### Spatial information relevant for local navigation

Whereas collision avoidance and landing are spatial tasks that must be solved by any flying insect, local navigation is relevant especially for particular insects, such as bees, some wasps and ants, which care for their brood and, thus, have to return to their nest after foraging. Consequently, the full complexity of spatial navigation has been analysed mainly in bees, wasps, and ants both in artificial and natural environments. Nonetheless, basic elements of local navigation could be found also in *Drosophila* (Foucaud et al., 2010; Ofstad et al., 2011). Since various aspects of insect navigation and the underlying mechanisms have been reviewed recently (Collett and Collett, 2002; Collett et al., 2006; Zeil et al., 2009; Zeil, 2012), only selected issues will be addressed here, and spatial information processing during flight will be the major focus.

Visual landmarks represent crucial spatial cues and are employed to localize a goal, especially if it is barely visible itself. Information about the landmark constellation around the goal is memorized during elaborate learning flights: the animal flies characteristic sequences of ever increasing arcs while facing the area around the goal. During these learning flights, the animal somehow gathers relevant information that is subsequently used to relocate the goal when returning to it after an excursion. A variety of visual cues, such as contrast, texture and color, are suitable to define landmarks and are employed to find the goal (reviews: Collett and Collett, 2002; Collett et al., 2006; Zeil et al., 2009; Zeil, 2012). Recently, landmarks that are defined by motion cues alone were shown to be sufficient for bees to locate the goal (Dittmar et al., 2010). In this study, several landmarks that were camouflaged by their texture and, thus, could not be discriminated from the background by stationary cues were placed in particular locations surrounding the goal (Figure 2C). The mechanisms by which the landmark constellation is learnt and how the memorized information is eventually used to locate the goal are not yet fully understood. However, it is clear that optic flow information generated actively during the bees' typical learning and searching flights is essential for the acquisition of a spatial memory of the goal environment. Moreover, in the vicinity of the landmarks, the animals were found to adjust their flight movements according to specific textural properties of the landmarks (Dittmar et al., 2010; Braun et al., 2012).

Landmarks close to the goal are, for geometrical reasons, most suitable to define the goal location, because the retinal locations of close landmarks are displaced more than distant ones during the translational movements of the animal (Stürzl and Zeil, 2007). Emerging as a direct consequence of the closed action–perception cycle, this property “weighs” the relevance of environmental objects to serve as landmarks for local navigation in the vicinity of the goal.

## Spatial information based on saccadic gaze and flight strategy

**Figure 3:**
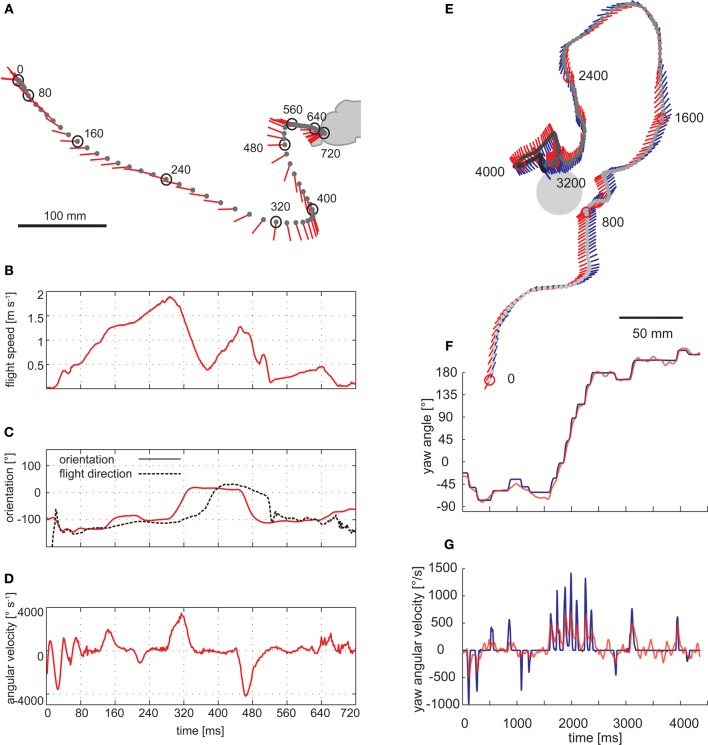
**Saccadic flight and gaze strategy of free-flying blowflies and honeybees. (A)** Sample flight trajectory of a blowfly as seen from above. The position of the fly (black dot) and the orientation of the longitudinal body axis (red line) are shown every 10 ms. The trajectory was filmed outdoors: the fly took off from a perch and landed on a leaf of a shrub. **(B)** Translational flight speed. **(C)** Orientation of the fly's longitudinal body axis (solid red line) and flight direction (broken black line) in the external coordinate system. **(D)** Angular velocity of the fly. The fly changed its gaze and heading direction through a series of short and fast body turns. Flight direction and body axis orientation frequently deviate: the body axis already points in the new flight direction while the fly is continuing to move on its previous course. **(A–D)** Data from Boeddeker et al. (2005). **(E)** Top view of a flight of a honeybee eventually landing on a feeder. The position of the bee's head (gray dot) is shown every 16 ms. The orientation of the head (blue line) and body (red line) can deviate considerably. **(F)** Head (blue) and body orientation (red). The head usually turns with the thorax but at a higher angular speed, starting, and finishing slightly earlier. **(G)** Head (blue) and body (red) angular velocity. **(E–G)** Data from Boeddeker et al. (2010).

Saccadic gaze changes have a rather uniform time course and are shorter than 100 ms. Angular velocities of up to several thousand °/s can occur during saccades (Figure 3). Since roll movements of the body that are performed for steering purposes during saccades, and also during sideways translations, are compensated by counter-directed head movements, the animals' gaze direction is kept virtually constant during intersaccades (Schilstra and van Hateren, 1999; Boeddeker and Hemmi, 2010; Boeddeker et al., 2010; Braun et al., 2010, 2012; Geurten et al., 2010, 2012). Saccade dynamics in flies have been shown to be fine-tuned by mechanosensory feedback from the halteres, the gyroscopic sense organs of dipteran flies, evolutionarily developed from the hind wings. Haltere feedback may thus contribute to increasing the duration of intersaccadic intervals (Sherman, 2003; Bender and Dickinson, 2006a). Nevertheless, halteres are no prerequisite for a saccadic gaze strategy, given that bees and wasps show similar flight dynamics as flies without halteres (Figure 3) (Boeddeker et al., 2010). By squeezing body and head rotations into the brief saccades, translational gaze displacements last for more than 80% of the entire flight time (van Hateren and Schilstra, 1999; Boeddeker and Hemmi, 2010; Boeddeker et al., 2010; Braun et al., 2010, 2012; van Breugel and Dickinson, 2012).

It should be noted that flying insects may appear to meander smoothly when their overall flight trajectory is inspected (Boeddeker et al., 2005; Kern et al., 2012). Having frequently been an issue of misunderstandings, this smoothness does not contradict a saccadic flight style. As a consequence of inertial forces, flying insects, in particular large ones, may move for some time after a saccadic change in body orientation in their previous direction. Thus, the saccadic gaze strategy is reflected only to some extent in the overall flight trajectories. (Figure 3). This may be different in the much smaller *Drosophila* where at least some rapid large-amplitude turns can be seen in the overall flight trajectories (Tammero and Dickinson, 2002b).

Blowflies do not fly exactly straight even in straight flight tunnels without any obstacles. Rather they perform sequences of saccades, alternating their direction and the saccade amplitude depending on the clearance of the animal with respect to the walls of the flight tunnel (Kern et al., 2012). A saccadic flight style may be functionally relevant, even if the overall flight course pursued by the animal is straight. This is because the animal normally has no prior knowledge about the spatial structure of the environment. Thus, the uncertainty about whether it can fly on a straight course or not needs to be resolved on the basis of optic flow information. Regular changes of flight and gaze direction might, therefore, be a useful flight strategy, because it would allow the animal to check (during intersaccadic intervals) the translational optic flow for environmental information (Kern et al., 2012).

Since the saccadic flight and gaze strategy leads to either primarily rotational or primarily translational optic flow on the eyes, it can be interpreted as a behavioral adaptation to facilitate spatial vision. This is because only translational optic flow depends on the distance of the animal to environmental objects and, thus, contains spatial information (see above). A segregation of optic flow fields into their rotational and translational components can, at least in principle, be accomplished computationally for most realistic situations (Longuet-Higgins and Prazdny, 1980; Prazdny, 1980; Dahmen et al., 2000). However, such a computational strategy for the nervous system appears to be a lot more demanding than preventing the formation of composite rotational and translational optic flow by behavioral means. Thus, a saccadic gaze and flight strategy can be regarded as an efficient way to provide the nervous system with input from which spatial information can be extracted with relatively little computational effort.

### Control of saccades as the main rotational components of flight behavior

The saccadic gaze strategy of insects has been characterized in various functional contexts: flies exhibit a saccadic flight pattern during spontaneous behavior, for instance, when cruising around without any obvious goal. This was shown in a wide range of environments including outdoors conditions (Figure 3A). Saccade frequencies of up to 10 per second were observed (Schilstra and van Hateren, 1999; van Hateren and Schilstra, 1999; Tammero and Dickinson, 2002b; Boeddeker et al., 2005, 2010; Braun et al., 2010, 2012; Dittmar et al., 2010; Geurten et al., 2010). The direction, amplitude and frequency of saccades depend not only on the spatial outline, but also on the texture of the environment. Thus, saccades are, at least to some extent, under visual control and serve purposes in spatial behavior, such as in collision avoidance behavior (Frye and Dickinson, 2007; Geurten et al., 2010; Braun et al., 2012; Kern et al., 2012).

There is consensus that intersaccadic optic flow during collision avoidance behavior plays a decisive role in controlling the direction and amplitude of saccades. However, which optic flow parameters may be most relevant is still inconclusive. Notwithstanding, all proposed mechanisms of evoking saccades rely on some sort of asymmetry in the optic flow pattern in front of the two eyes. The asymmetry may be due to the location of the expansion focus in front of one eye or to a difference between the overall optic flow in the visual fields of the two eyes (Tammero and Dickinson, 2002b; Lindemann et al., 2008; Mronz and Lehmann, 2008; Kern et al., 2012).

Not all of the visual field has been concluded to be involved in saccade control, at least for blowflies. The optic flow in the lateral parts of the visual field does not play a role in determining saccade direction (Kern et al., 2012). This feature might be related to the way in which blowflies fly: during intersaccades, they predominantly fly forwards with some sideways component after saccades that shifts the pole of expansion of the flow field slightly toward frontolateral locations (Kern et al., 2012). In contrast, in *Drosophila*—which are able to hover and fly sideways (Ristroph et al., 2009)—lateral and even rear parts of the visual field have also been shown to be involved in saccade control. Therefore, in *Drosophila*, a mechanism that also takes lateral retinal areas into account for saccade control is plausible from a functional point of view (Tammero and Dickinson, 2002b).

### Control of intersaccadic translational motion

Whereas saccades are fairly stereotyped across different behavioral contexts, the intersaccadic translational movements may vary to a much larger extent, depending on the behavioral context as well as the spatial layout of the environment (Braun et al., 2010, 2012). This aspect has been addressed systematically in two different behavioral contexts: (1) The dependence of translation velocity on the spatial layout of the environment, and (2) the control of translational movements during visual landmark navigation in the vicinity of an invisible goal.

**Figure 4:**
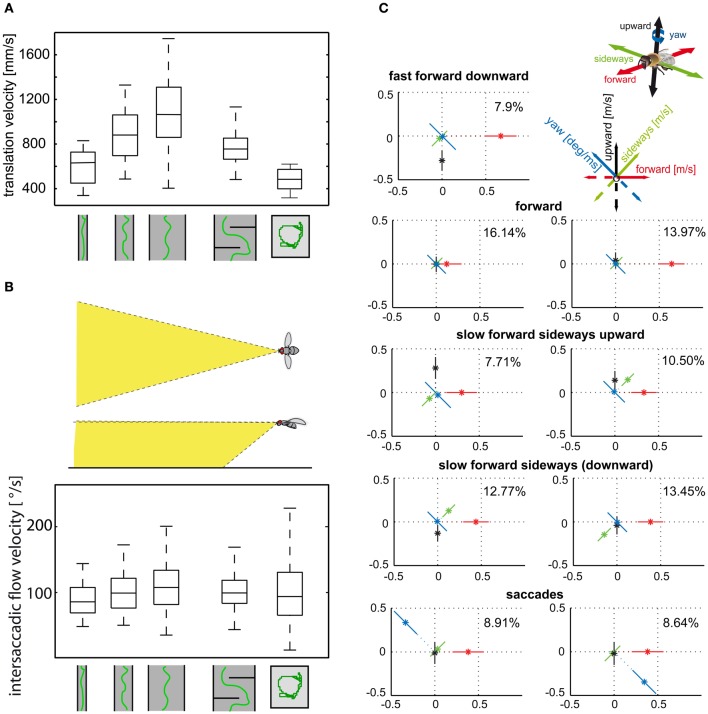
**(A)** Control of translational velocity in blowflies. Boxplot of the translational velocity in flight tunnels of different widths, in a flight arena with two obstacles and in a cubic flight arena (sketched below data). Translation velocity strongly depends on the geometry of the flight arena. **(B)** Boxplot of the retinal image velocities within intersaccadic intervals experienced in the fronto-ventral visual field (see inset) in the different flight arenas. In this area of the visual field, the intersaccadic retinal velocities are kept roughly constant by regulating the translation velocity according to clearance with respect to environmental structures. The upper and lower margins of the boxes in **(A)** and **(B)** indicate the 75th and 25th percentiles, and the whiskers the data range (Data from Kern et al., 2012). **(C)** Translational and rotational prototypical movements of honeybees during local landmark navigation (see example in Figure 2C). Homing flight sequences can be decomposed into nine prototypical movements using clustering algorithms in order to reduce the behavioral complexity. Each prototype is depicted as a star plot containing the four velocity components drawn onto color-coded lines equally dividing the drawing plane (see inset). For each line, the distance of the dot from the center determines the value of the corresponding velocity component, and the error bars give the standard deviation of this value. Percentage values provide the relative occurrence of each prototype. More than 80% of flight-time corresponds to a varied set of translational prototypical movements and less than 20% has significantly non-zero rotational velocity corresponding to the saccades (Data from Braun et al., 2012).

Insects tend to decelerate when their flight path is obstructed. Flight speed is thought to be controlled by optic flow generated during translational flight (David, 1979, 1982; Farina et al., 1995; Kern and Varjú, 1998; Baird et al., 2005, 2006, 2010; Frye and Dickinson, 2007; Fry et al., 2009; Dyhr and Higgins, 2010; Straw et al., 2010; Kern et al., 2012). Flies, bees, and moths were concluded to keep the optic flow on their eyes at a “preset” total strength by adjusting their flight speed. Accordingly, they decelerate when the translational optic flow increases, for instance, while passing a narrow gap or flying in a narrow tunnel (Figures 4A,B) (Srinivasan et al., 1991, 1996; Verspui and Gray, 2009; Baird et al., 2010; Portelli et al., 2011; Kern et al., 2012). However, not all parts of the visual field contribute equally to the input of the velocity controller. Whereas the intersaccadic optic flow generated in eye regions looking well in front of the insect has a strong impact on flight speed, the lateral visual field plays only a minor role (Baird et al., 2010; Portelli et al., 2011; Kern et al., 2012).

Translational flight maneuvres during the spatial navigation of bees have a particularly elaborate fine structure and can be described by a distinct set of prototypical movements (Figure 4C). The optic flow generated during flight sequences close to visual landmarks appears to be systematically employed to localize a virtually invisible goal. Not only the overall velocity, but also the relative distribution of sideways and forward translational movements depend on the insect's distance and orientation relative to the landmarks and the goal (Zeil et al., 2009; Dittmar et al., 2010, 2011; Braun et al., 2012; Zeil, 2012). Bees, for example, frequently tend to perform translational movements with a strong sideways component close to landmarks, as if they wanted to scrutinize them in detail. These sideways movements are more pronounced if the landmarks are camouflaged by the same texture as their background and, thus, can be detected only by relative motion cues in the optic flow fields (Dittmar et al., 2010; Braun et al., 2012).

## Processing of optic flow in the insect nervous system

Separating the rotational and translational optic flow components behaviorally can be viewed as an efficient strategy to reduce the computational load for the nervous system when extracting information about the environment and, especially, about its spatial layout. Nonetheless, the retinal image flow resulting from the closed action–perception cycle still has complex spatiotemporal properties, and its processing represents a demanding challenge for the nervous system. In particular, there is not much time for gathering environmental information between saccades. With up to 10 saccades per second being generated, intersaccadic intervals may be as short as only a few ms and rarely longer than 100–200 ms. Time is a critical issue for at least three reasons: (1) All neural processing is time-consuming, beginning with the biophysical mechanisms of signal transduction in the photoreceptors, and ending with transmitter signaling at neuromuscular junctions. (2) Sensory input is encoded by nerve cells with only limited reliability. Repeated presentation of the same input may lead to variable neural responses, which constrain the information which can be transmitted within a given time interval. (3) Neural computations are not necessarily rigid, but may flexibly adjust to the prevailing stimulus conditions. To be functionally beneficial, the time constants of such adaptive processes need to match the behaviorally relevant timescale of changes of the various visual stimulus parameters.

These three issues become particularly challenging if information is to be processed and represented with sufficient reliability on the very short timescales that are behaviorally relevant for fast flying insects. The virtuosity of the spatial behavior of many insects is proof that their sensory and nervous systems somehow cope successfully with this challenge. Since insects accomplish all this with very small brains comprising only a million or less neurons, they seem to be champions of resource efficient information processing and behavioral control.

So far, we only have vague conceptions of how all this is accomplished. In the following, we briefly sketch the available knowledge about the processing of retinal image flow. Particular focus is placed on how the spatiotemporal properties of image flow are shaped by the closed action–perception cycle.

### Spatiotemporal visual input of insects is shaped by active gaze strategies

From what has been sketched above, it may be obvious that the spatiotemporal characteristics of the input to the visual system will depend strongly not only on the features of the behavioral surroundings, but also on the specific dynamical characteristics of locomotion. These movements, resulting from the closed-loop nature of the behavior, may, in turn, depend on the environmental properties. The statistical properties of a wide variety of natural scenes have been characterized in many studies. The scenes analysed were usually stationary, or they resulted from movements either at constant velocities or with dynamics that differ a lot from that of unrestrained gaze changes during natural locomotion (e.g., Eckert and Buchsbaum, 1993; Dong and Attick, 1995; van Hateren, 1997; Simoncelli and Olshausen, 2001; Betsch et al., 2004; Geisler, 2008). In a recent study, we simulated the natural dynamics of the saccadic gaze strategy of insects and registered the resulting image sequences in a large variety of natural environments (Schwegmann et al., in preparation).

**Figure 5:**
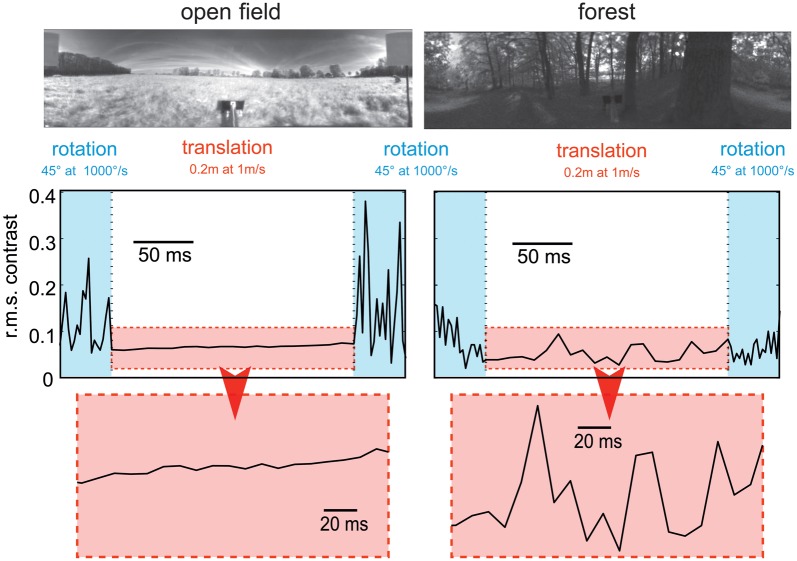
**Consequences of flight dynamics for contrast fluctuations in a small patch (2 × 2°, corresponding to approximately the aperture of a local movement detector) of the visual field at the equator and 90° relative to the direction of motion in two typical environments: an open field (left column) and a forest (right column).** The movement sequence of the panoramic camera system corresponds to an initial 45° rightward rotation at a saccade-like angular velocity (1000°/s), followed by a translation for 20 cm at a velocity of 1 ms and then another 45° rightward turn. In general, contrast fluctuations are much larger during saccade-like turns than during translational phases. If environmental objects are relatively close (as in the forest environment), translations may also lead to considerable contrast fluctuations, though on a slower timescale. The data are based on high dynamic range image sequences, which are rescaled to the printable contrast range. (Data from Schwegmann et al., in preparation).

Given the characteristic temporal structure of behavioral dynamics, the parameters within these image sequences also change in a temporally structured way. Two aspects of such changes may be particularly relevant for extracting behaviorally relevant environmental information from the retinal image flow: (1) Relevant image parameters, such as brightness, contrast, and spatial frequency composition, vary according to image region and viewing direction, and fluctuate more rapidly during saccadic turns than during intersaccades. (2) During translatory intersaccadic movements, image parameters resulting from close structures fluctuate in general much more than those resulting from distant structures (Figure 5).

The dynamical properties imposed by the saccadic gaze change and the image statistics of natural environments constrain the time constants of information processing. Furthermore, the adaptive mechanisms that are thought to adjust the sensitivity of the visual system to the prevailing stimulus conditions have to operate on a suitable timescale. In particular, to optimize the encoding of the fluctuations of environmental image features during the intersaccadic intervals, adaptation in the visual system should essentially take place on a timescale shorter than the duration of these intervals (i.e., within some tens of milliseconds) and may be driven by the high-frequency changes of the respective image parameters. Several physiological components of motion adaptation have been described at the different levels of the fly visual system (e.g., Maddess and Laughlin, 1985; Brenner et al., 2000a; Harris et al., 2000; Fairhall et al., 2001; Kurtz, 2007; Kalb et al., 2008; Liang et al., 2008). To what extent the time constants of these processes, which have been identified with experimenter designed motion stimuli, match the dynamics of parameter changes in the natural visual input, and how these adaptive processes are controlled, is still not clear.

### Peripheral processing of motion information

How is the environmental and, in particular, the spatial information extracted from the retinal image flow and represented in the visual motion pathway? The retinal input is transformed at the level of photoreceptors in basically two ways: (1) The retinal input is sampled by the array of photoreceptors. Compared with technical imaging systems, the number of image points and, thus, the spatial resolution is very low, with only approximately 750 image points per eye in *Drosophila* (Hardie, 1985), 5000 in the blowfly *Calliphora* (Beersma et al., 1977) and 5400 in honeybees (Seidl and Kaiser, 1981). The visual angle between photoreceptors is matched by their acceptance angle resulting in a blurred retinal image (Götz, 1965; van Hateren, 1993). Despite the low spatial resolution of the eyes of insects, they are obviously able to accomplish even intricate spatial vision tasks (see above). The low number of retinal input channels reduces the computational load for subsequent information processing tremendously and, thus, may be one reason why insects are so efficient with respect to computational expenditure. (2) As a consequence of the biophysical transduction machinery, the photoreceptors represent a kind of temporal low-pass filter. Owing to adaptive mechanisms, the strength of this temporal blurring depends on the ambient brightness, with the time-constants of blurring reflecting a trade-off between fast transmission and the reliability of the retinal output signals given the stochastic nature of the photons impinging on the photoreceptors (Juusola et al., 1994, 1996; Juusola, 2003).

**Figure 6:**
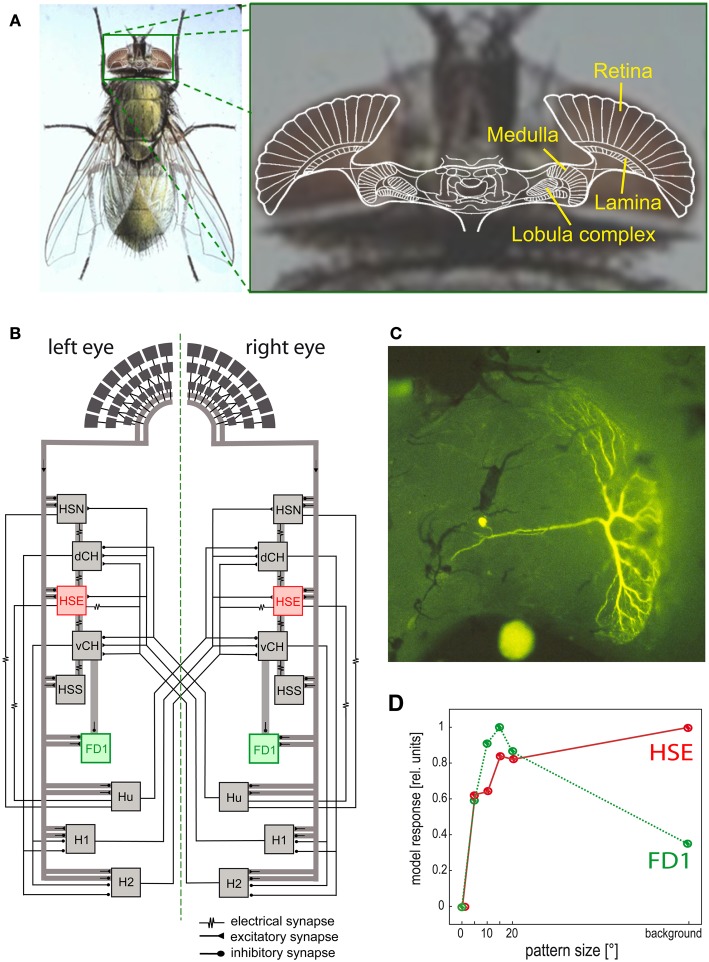
**Visual system of the blowfly and neural circuits extracting optic flow information from the retinal image sequences. (A)** Schematic of a horizontal section of the fly's brain projected onto a photograph of its head, with the retina and the three main visual neuropiles labeled. **(B)** Wiring sketch of some LWCs sensitive to different types of horizontal motion in the lobula plate of the blowfly. The HSE cells, one type of HS cells, which respond best to coherent wide-field motion, and the FD1 cells, a special type of FD cells, which are most sensitive to the motion of objects, are highlighted. **(C)** Structure of an FD1 cell with its dendritic tree residing in the lobula plate. The cell is shown in a whole-mount preparation after it has been injected with the fluorescent dye Lucifer Yellow. **(D)** Dependence of the normalized response amplitude of an HSE and a FD1 cell on the angular horizontal extent of a moving pattern. The responses are based on computer simulations of a circuit model [as shown in **(B)**]; the model responses mimic the physiologically determined responses (Data from Hennig and Egelhaaf, 2012).

The photoreceptor output is fed into the neural network of the first visual neuropile, the lamina (Figure 6A). Here, those photoreceptors looking at the same point in visual space converge on common second order neurons (Kirschfeld, 1972), thereby increasing the reliability of signal transmission, especially at low-light intensities (Laughlin, 1994). The photoreceptor signals are further processed in the lamina. (1) They are temporally band-pass filtered, thereby enhancing the representation of contrast changes in the retinal images (Laughlin, 1994; van Hateren, 1997). Owing to the special properties of the synapses between photoreceptors and second order neurons, the signal time course becomes faster and more transient with increasing background intensity (Juusola et al., 1995). Given the noisiness of the input signals and the limited dynamic range of nerve cells, the overall brightness-dependent spatiotemporal filter properties of the peripheral visual system are thought to maximize the flow of information about natural moving images (van Hateren, 1992). It should be noted that these conclusions are based so far on image sequences resulting from smoothly superimposed rotational and translational movements, without taking the different dynamical properties of image changes during saccades and intersaccades into account. During translational intersaccadic movements, the image dynamics can be expected to depend on the depth structure of the scenery, because the retinal images of distant objects move at lower velocities than those of near objects (Figure 5). (2) Recent evidence based on targeted genetic manipulations of individual cell types in the peripheral visual system of *Drosophila* indicate, though there are differences in details between studies, that the lamina output is segregated into parallel ON and OFF pathways, signaling either brightness increases or decreases (Joesch et al., 2010; Reiff et al., 2010; Clark et al., 2011). One functional consequence of splitting the visual input into ON and OFF components is to facilitate the biophysical implementation of the mechanism of motion detection at subsequent stages of the visual system. The core of this mechanism is a multiplication-like interaction between neighboring retinal input channels (see below), which gives a positive output for two positive as well as for two negative inputs (Egelhaaf and Borst, 1992, 1993b; Eichner et al., 2011).

### Local motion computation

A lot is known, especially in flies, about the computations underlying motion vision. The available evidence on bees suggests that motion information is processed in their visual system according to similar principles. Local motion detection is assumed to be accomplished in the second visual neuropile, the medulla (Figure 6A). Motion-specific responses have been found in the two most proximal layers of the medulla. Most motion sensitive medulla neurons that could be functionally characterized have small receptive fields, as is expected from neurons involved in local motion detection (review: Strausfeld et al., 2006). As a consequence of the small size of the neurons in this brain area and the difficulty of recording their activity, conclusions concerning the cellular mechanisms underlying motion detection are still tentative. A lot of progress is currently being made by combining the sophisticated repertoire of genetic and molecular approaches in *Drosophila* with electrophysiological and imaging techniques to identify the different components of the neural circuits underlying motion detection (Rister et al., 2007; Joesch et al., 2008, 2010; Katsov and Clandinin, 2008; Borst, 2009; Reiff et al., 2010; Clark et al., 2011; Schnell et al., 2012).

A large number of features of motion detection can be accounted for by a computational model, the so-called correlation-type motion detector. In its simplest form, a local motion detector is composed of two mirror-symmetrical subunits. In each subunit, the signals of adjacent light-sensitive cells receiving the filtered brightness signals from neighboring points in visual space are multiplied after one of them has been delayed. The final detector response is obtained by subtracting the outputs of two such subunits with opposite preferred directions, thereby considerably enhancing the direction selectivity of the motion detection circuit. Each motion detector reacts with a positive signal to motion in a given direction and with a negative signal to motion in the opposite direction (reviews: Reichardt, 1961; Borst and Egelhaaf, 1989; Egelhaaf and Borst, 1993b). Various elaborations of this basic motion detection scheme have been proposed to account for the responses of insect motion-sensitive neurons under a wide range of stimulus conditions including even natural optic flow as experienced under free-flight conditions (see e.g., Borst et al., 2003; Lindemann et al., 2005; Brinkworth et al., 2009).

### Extraction of optic flow information

Since the optic flow as induced during locomotion has a global structure, it cannot be represented in any specific way by local mechanisms alone. Rather, local motion measurements from large parts of the visual field need to be combined. This is accomplished in the third visual neuropile, the lobula complex, by directionally selective wide-field neurons (Figure 6) in all insect species analysed so far. Independent of the species under investigation, these neurons will here be collectively referred to as LWCs (lobula complex wide-field cells). LWCs have been investigated in particular detail in flies, where they reside in the distinct posterior part of the lobula complex; they are, therefore, often termed lobula plate tangential cells (LPTCs). In bees, the lobula complex is undivided; however, bees have very similar motion-sensitive wide-field neurons to those characterized in the lobula plate of flies (DeVoe et al., 1982; Ibbotson, 1991). Most LWCs spatially pool the outputs of many retinotopically arranged local motion-sensitive neurons on their large dendrites and, accordingly, have large receptive fields. These local motion-sensitive neurons are thought to correspond to the local motion detectors, as described above. LWCs are excited by motion in their preferred direction and are inhibited by motion in the opposite direction (reviews: Hausen and Egelhaaf, 1989; Krapp, 2000; Borst and Haag, 2002; Egelhaaf et al., 2002; Egelhaaf, 2006; Taylor and Krapp, 2008; Borst et al., 2010).

For fly LWCs, the local motion-sensitive elements that synapse onto their dendrites have been concluded to differ in their preferred direction of motion. As a consequence, local preferred directions of LWCs change gradually over their receptive field and it has been suggested that they coincide with the directions of the velocity vectors characterizing the flow fields that are induced during certain types of self-motion (Hausen, 1982; Krapp et al., 1998, 2001; Petrowitz et al., 2000; Taylor and Krapp, 2008).

Despite the characteristic patterns of preferred directions in the receptive fields of LWCs, dendritic pooling of motion input is not sufficient to obtain specific responses during particular types of self-motion. Network interactions, mediated by both electrical and chemical synapses, between LWCs within one brain hemisphere and between both halves of the visual system are important for shaping their specific sensitivities for optic flow (Figure 6B; reviews: Borst and Haag, 2002; Egelhaaf et al., 2002; Egelhaaf, 2006; Borst et al., 2010). To enhance the specificity of LWCs for particular global optic flow patterns, interactions between both visual hemispheres are particularly relevant. The optic flow, for instance, across both eyes during forward translation is directed backwards. In contrast, during a pure rotation about the animal's vertical axis, optic flow is directed backwards across one eye, but forwards across the other eye. Thus, translational and rotational optic flow can, at least in principle, be distinguished if motion from both eyes is taken into account (Hausen, 1982; Egelhaaf et al., 1993; Horstmann et al., 2000; Farrow et al., 2003, 2006; Karmeier et al., 2003; Borst and Weber, 2011; Hennig et al., 2011). Other LWCs of blowflies, the figure detection (FD) cells, respond best to the motion of objects rather than to global optic flow patterns. This object sensitivity could be shown for one prominent element of this group of cells to be a consequence of inhibitory synaptic interactions with other LWCs (Figures 6B–D) (Egelhaaf, 1985b; Warzecha et al., 1993; Kimmerle and Egelhaaf, 2000a,b; Hennig et al., 2008, 2011; Hennig and Egelhaaf, 2012; Liang et al., 2012). FD cells are thought to play a prominent role in detecting stationary objects in the environment, such as landing sites that are distinguished from their background by motion, and also other visual cues. Other LWCs found in various fly species respond to much smaller objects than do FD cells. These cells were interpreted as being involved in detecting and pursuing prey and/or mates (Olberg, 1981, 1986; Gilbert and Strausfeld, 1991; Nordström et al., 2006; Nordström and O'Carroll, 2006; Barnett et al., 2007; Geurten et al., 2007; Trischler et al., 2007) and it is suggested they owe their exquisite sensitivity for extremely small targets to a variety of local and global synaptic interactions (Nordström, 2012).

Although the synaptic interactions between LWCs may increase their specificity for particular types of optic flow and stimulus sizes, this specificity is usually far from being perfect, and most neurons still respond to a wide range of “non-optimal” stimuli indicating that behaviorally relevant motion information is encoded by the activity profile of populations of LWCs rather than by the responses of individual cells.

Despite their specific differences, LWCs have general properties which may be functionally relevant in the context of spatial vision.

**Figure 7:**
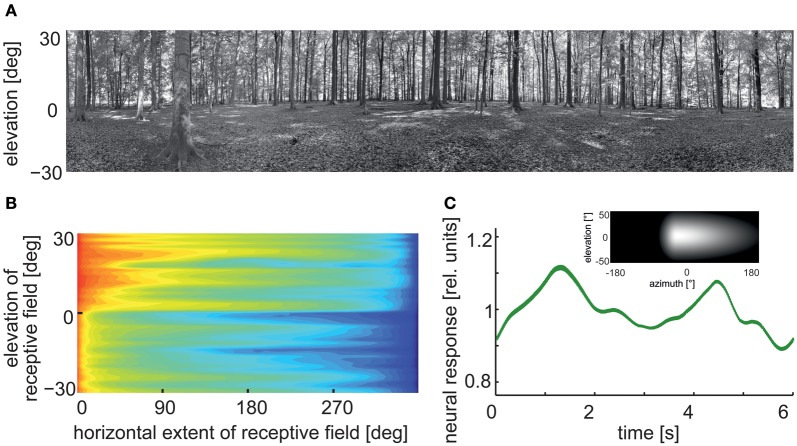
**Pattern-dependent response modulations of modeled arrays of movement detectors. (A)** Panoramic high dynamic range image of a forest scene (rescaled in contrast for printing purposes). **(B)** Logarithmic color-coded standard deviation describing the mean pattern-dependent modulations for one-dimensional receptive fields differing in the elevation of receptor position and azimuthal receptive field size. Pattern-dependent modulations decrease with horizontal receptive field extent. Modulation amplitude depends on the contrast distribution of the input image, as can be seen when comparing pattern-dependent modulation amplitudes corresponding to the upper (trees) and lower part (ground) of the input image. **(C)** Time-dependent response of an array of movement detectors with an estimated HSE cell receptive field. Inset: Weight field of the spatial sensitivity distribution of a HSE cell. The brighter the gray level, the larger the local sensitivity. The frontal equatorial viewing direction is at 0° azimuth and 0° elevation. Image motion was performed for 12 s in the preferred direction of the model cell at an angular velocity of 60°/s (Data from Meyer et al., 2011).

- *Velocity dependence*: LWCs do not operate like odometers: their mean responses increase with increasing velocity, reach a maximum, and then decrease again. Hence, their response does not reflect pattern velocity unambiguously. This ambiguity is even more complex, since the location of the velocity maximum depends on the textural properties of the moving stimulus pattern. If the spatial frequency of a drifting sine-wave grating is shifted to lower values, the velocity optimum shifts to higher values. In terms of the correlation model of motion detection, the location of the temporal frequency optimum is determined by the time constant of the delay filters in the local motion detectors (review: Egelhaaf and Borst, 1993b). The pattern dependence of velocity tuning is reduced if the stimulus pattern consists of a broad range of spatial frequencies, as is characteristic of natural scenes (Dror et al., 2001; Straw et al., 2008). Despite these ambiguities, flies and bees appear to regulate their intersaccadic translation velocity during free-flight to keep the retinal velocities in that part of the operating range of the motion detection system in which responses increase monotonically with retinal velocities (Baird et al., 2010; Portelli et al., 2011; Kern et al., 2012).
- *Time course of motion responses*: The representation of image velocity becomes even more complex if we take time-varying pattern velocities into account, as are characteristic of behavioral situations. The time course of LWC responses is roughly proportional to pattern velocity only as long as the velocity changes are small (Egelhaaf and Reichardt, 1987; Haag and Borst, 1997, 1998). However, as a consequence of the computational structure of local motion detectors, LWC responses do not only depend on pattern velocity, but also on higher-order temporal derivatives (Egelhaaf and Reichardt, 1987). This is reflected, for instance, in the response transients to sudden changes in pattern velocity (Egelhaaf and Borst, 1989; Egelhaaf and Warzecha, 1998; Warzecha et al., 1998). The rapid saccadic turns characterizing insect free-flight probably lead to the most transient retinal image displacements that occur under natural conditions. The retinal peak velocities attained during saccades of up to several thousands of degrees per second are far beyond the velocity optima determined even for transient conditions (Maddess and Laughlin, 1985; Warzecha et al., 1999). Nonetheless, saccade direction can be encoded by LWCs by transient responses with corresponding signs. However, this is the case only as long as the cell is not excited by translational optic flow during intersaccades, for example, when the animal flies close to environmental structures. In this case, the cell may be depolarized more strongly by the translational optic flow than by a preferred-direction saccade, even though the translational velocities are much smaller than the velocities evoked by the saccades (Kern et al., 2005; van Hateren et al., 2005).
- *Motion adaptation*: Motion vision systems operate under a variety of dynamical conditions. Accordingly, several response features of LWCs have been shown to depend on stimulus history in a characteristic way. A number of mechanisms are involved in the corresponding changes in the visual motion pathway. Some of them operate locally and, thus, presynaptic to the LWCs; they are, to some extent, independent of the direction of motion. Other mechanisms originate after spatial pooling of local motion signals at the level of LWCs, making them dependent on the direction of motion (reviews: Clifford and Ibbotson, 2003; Egelhaaf, 2006; Kurtz, 2009). All these processes are usually regarded as adaptive, although their functional significance is still not entirely clear. Several non-exclusive possibilities have been proposed, such as adjusting the dynamic range of motion sensitivity to the prevailing stimulus dynamics (Brenner et al., 2000a; Fairhall et al., 2001), saving energy by adjusting the neural response amplitudes without affecting the overall information that is conveyed (Heitwerth et al., 2005), and increasing the sensitivity to changes in stimulus parameters resulting from environmental discontinuities (Maddess and Laughlin, 1985; Liang et al., 2008, 2011; Kurtz et al., 2009).
- *Gain control by dendritic integration of antagonistic motion input*: Dendritic integration of signals from local motion-sensitive elements by LWCs is a highly non-linear process. When the signals of an increasing number of input elements are pooled, saturation non-linearities make the response largely independent of pattern size. However, the response saturates at different levels for different velocities. Hence, LWC responses are almost invariant against changes in pattern size, while they still depend on velocity. This gain control can be explained on the basis of the passive membrane properties of LWCs and the antagonistic nature of their motion input. Even motion in the preferred direction activates both types of the two mirror-symmetrical subunits of the motion detector, for instance, excitatory and inhibitory inputs of LWCs, though to a different extent, depending on the velocity of motion. As a consequence, the saturation levels reached by the membrane potential of an LWC with increasing numbers of activated input elements are different for different velocities (Hausen, 1982; Egelhaaf, 1985a; Borst et al., 1995; Single et al., 1997).
- *Pattern dependence*: The responses of the local input elements of LWCs are temporally modulated even during pattern motion at a constant velocity owing to their small receptive fields. These modulations are the consequence of the texture of the environment. Since the signals of neighboring input elements are phase-shifted with respect to each other, their pooling by the dendrites of LWCs reduces mainly those pattern-dependent response modulations that originate from the high spatial frequencies of the stimulus pattern. The pattern-dependent response modulations decrease with the increasing size of the receptive field (Figure 7) depending, to some extent, on its aspect ratio (Egelhaaf et al., 1989; Single and Borst, 1998; Dror et al., 2001; Meyer et al., 2011; O'Carroll et al., 2011; Hennig and Egelhaaf, 2012; Kurtz, 2012). From the perspective of velocity coding, the pattern-dependent response modulations have been viewed as “pattern noise” because they deteriorate the quality of the neural representation of pattern velocity (Dror et al., 2001; O'Carroll et al., 2011). Alternatively, these pattern-dependent modulations may be functionally relevant, as they reflect the textural properties of the surroundings (Meyer et al., 2011; Hennig and Egelhaaf, 2012). We will argue below that the latter interpretation might be relevant especially during translatory locomotion during intersaccadic intervals.

## Behavioral significance of optic flow neurons

What is the functional significance of the response characteristics of the motion sensitive and directionally selective LWCs described above? Two related and, to some extent, interdependent views are prevalent in the literature: (1) LWCs are conventionally conceived as self-motion sensors and, in particular, rotation detectors, in other words, neural elements sensing deviations of the animal from its normal attitude and/or flight course. (2) It is often implicitly assumed that the motion detection system should produce responses that come close to a veridical representation of the retinal velocities. Deviation from this velocity representation, such as the ambiguities in the responses resulting from the pattern properties of the stimulus and the fact that the response first increases with increasing velocity, but then decreases again beyond some velocity level (see above), are then regarded as deficiencies of an imperfect biological mechanism. However, it is becoming increasingly obvious from recent research that both views need to be qualified given the peculiar spatiotemporal characteristics of the retinal image flow resulting from the active vision strategies of insects. Moreover, constraints imposed by the timescale of behavior need to be taken into account when interpreting the functional significance of LWCs.

### A role of LWCs in mediating compensatory optomotor turning responses

LWCs are commonly thought to mediate compensatory optomotor turning responses of the entire body as well as the head. The strongest, though not very specific, evidence is based on the fact that many characteristics of the behavioral responses correlate well with the response characteristics of LWCs: they show similar velocity sensitivity, and the local preferred directions of various LWCs appear to match with rotational optic flow fields and, thus, were interpreted as an adaption to detect rotational self-motion of the animal around different axes (Krapp and Hengstenberg, 1996; Krapp et al., 1998, 2001; Krapp, 2000; Elyada et al., 2009).

Optomotor following of the entire animal is often analysed in tethered flight both under open- and closed-loop conditions: Here, the fly generates turning responses of the head and the body and follows the moving pattern. This response is usually interpreted to reflexively stabilize the retinal images by minimizing the retinal velocities, for instance, resulting from external and/or internal disturbances (Hausen and Egelhaaf, 1989; Krapp, 2000; Borst and Haag, 2002; Egelhaaf, 2006; Taylor and Krapp, 2008; Borst et al., 2010). However, only rotational optic flow can be eliminated in this way, and the retinal images cannot be stabilized entirely during flight, because the animal needs to translate if it wants to move from one place to another.

A general feature of compensatory optomotor responses is that they are relatively slow. Their response dynamics differ considerably from the much faster object-induced fixation responses (Egelhaaf, 1987, 1989; Warzecha and Egelhaaf, 1996; Duistermars et al., 2007; Rosner et al., 2009). What is the functional significance of such slow compensatory optomotor responses under natural behavioral conditions? Since intersaccadic gaze stabilization is very fast, it is hardly conceivable that it could be controlled by optomotor feedback. Optomotor feedback can play a role only at a much slower timescale, for instance, to compensate for steady asymmetries at the level of the sensory input (e.g., dirt on one eye or internal gain differences) or the motor output (e.g., worn-out wings). Evidence for this comes from experiments where asymmetries were introduced to the visual system by occluding one of the eyes (Kern et al., 2000, in preparation). These behavioral results indicate that LWCs may play a role in mediating compensatory responses of the animal to slow unintended deviations from course, after their output signals are considerably low-pass filtered. So far, it is not clear where in the nervous system downstream of the lobula complex and by what mechanisms this filtering is accomplished.

In addition to the body, the head of flies and bees also performs compensatory optomotor responses in both tethered and free-flight. Compensatory head movements are most prominent during roll rotations of the body as are generated during banked saccadic turns and during sideways translations (Hengstenberg, 1993; van Hateren and Schilstra, 1999; Boeddeker and Hemmi, 2010; Boeddeker et al., 2010; Geurten et al., 2010). Fast gaze stabilization in flies is mainly achieved by mechanosensory input from halteres that act as gyroscopes (Sandeman and Markl, 1980). However, some LWCs have a rather direct impact on head muscles and, thus, on mediating head rotations (Milde et al., 1987, 1995; Gronenberg and Strausfeld, 1990; Gronenberg et al., 1995; Huston and Krapp, 2008, 2009). Bees, like most other insects, lack specialized inertial sensors like halteres. Nonetheless, they also show an optomotor reflex that uses visual motion to stabilize the head with respect to the visual environment under free-flight conditions at retinal velocities of up to 300°/s (Boeddeker and Hemmi, 2010). Experiments on fruit flies provide a similar picture: whereas the visual system is tuned to relatively slow rotation, the haltere-mediated response to mechanical oscillation increases with rising angular velocity (Hengstenberg, 1993; Sherman and Dickinson, 2003, 2004).

In conclusion, LWCs are likely to mediate optomotor responses on a relatively slow timescale, and might thus help compensating rotational optic flow arising from internal asymmetries of the animal. Given the extremely rapid timescale on which gaze direction is stabilized during saccadic flight maneuvres and the response latencies of visually mediated head responses, the functional role of LWCs for compensatory head rotations under free-flight conditions is still not entirely clear.

### A role of LWCs in gathering information about the environment during intersaccadic intervals

The time that flies and bees keep their gaze straight amounts to more than 80% of the overall flight-time (Schilstra and van Hateren, 1999; van Hateren and Schilstra, 1999; Boeddeker et al., 2005, 2010; Braun et al., 2010, 2012; Geurten et al., 2010; van Breugel and Dickinson, 2012). Hence, rotations are squeezed into relatively short and rapid saccadic turns. This flight and gaze strategy has been interpreted as a way to facilitate gathering environmental information that is contained in the retinal image flow during translatory self-motion (see above). Therefore, motion-sensitive neurons appear to be predestined to provide environmental information during intersaccadic intervals.

This suggestion is plausible, because the specificity of most LWCs for rotational optic flow is not exclusive and they also respond strongly to translational optic flow (Hausen, 1982; Horstmann et al., 2000; Karmeier et al., 2003, 2006; Taylor and Krapp, 2008). Moreover, the most prominent rotations performed by insects in free-flight, the saccadic turns, lead to angular velocities that are much beyond the monotonic operating range of the motion detection system (see above); rather the monotonic operating range roughly matches the intersaccadic translational velocities in those retinal regions that are probably involved in controlling the translation velocity of the animal (Kern et al., 2012).

**Figure 8:**
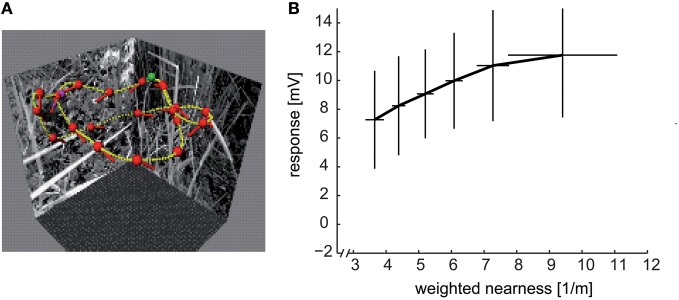
**Distance dependence of intersaccadic responses in the HSE cell, a prominent LWC in the blowfly lobula plate. (A)** Sample flight trajectory of a blowfly in a cubic arena used for the reconstruction of optic flow. The track of the fly is indicated by the yellow lines; red dots and short dashes indicate the position of the fly's head and its orientation, respectively; green and violet dots indicate the start and end of the trajectory, respectively. **(B)** Average intersaccadic responses of HSE cell recordings from three different flight trajectories plotted versus the corresponding average weighted nearness. The responses were sorted by increasing nearness and then attributed to six groups. The vertical and horizontal lines show the standard deviations of responses and nearness, respectively, across the data values within one group. The intersaccadic responses were related to the nearness of the fly to the respective arena walls (nearness = 1/distance), weighted by the HSE cell's spatial sensitivity distribution (see inset of Figure 7C). The intersaccadic responses increase with increasing nearness to the walls of the flight arena (Data from Liang et al., 2012).

As has been stressed above, LWCs are not veridical sensors of velocity and, thus, do not provide unambiguous information about self-motion. This is particularly obvious for the translatory movements during intersaccadic intervals, because here, retinal velocities do not only depend on the velocity of locomotion, but also on the three-dimensional layout of the environment. This dependency is reflected in the responses of HS cells; a group of three fly LWCs with a main preferred direction from the front to the back in the visual field of one eye. These neurons depolarize if environmental structures are sufficiently close, especially during translatory self-motion with a strong sideways component (Figure 8) (Boeddeker et al., 2005; Kern et al., 2005; Lindemann et al., 2005; Liang et al., 2012). Similar results were obtained in further LWCs during translatory movements in other directions (Karmeier et al., 2006). However, spatial information is only provided by LWCs if rotational movements are largely eliminated during the intersaccadic intervals, emphasizing the importance of the active saccadic flight and gaze strategy in the context of spatial vision (Kern et al., 2006). The responses to objects nearby are even more augmented by adaptation mechanisms, which depend on stimulus history, and, thus, on the properties of previous flight sequences (Liang et al., 2008, 2011).

What is the range within which spatial information is encoded in this way? Under spatially constrained conditions where the flies flew at translational velocities of only slightly more than 0.5 metres per second, the spatial range within which significant distance dependent intersaccadic responses are evoked amounts to approximately two metres (Kern et al., 2005; Liang et al., 2012). Since a given retinal velocity is determined in a reciprocal way by distance and velocity of self-motion, respectively, the spatial range that is represented by LWCs can be expected to increase with increasing translational velocity. In other words, the behaviorally relevant spatial range can be assumed to scale with locomotion velocity. From an ecological point of view, this consequence of the closed-loop nature of vision is economical and efficient, since the behaviorally relevant spatial depth range increases during fast self-motion. A fast moving animal can thus initiate an avoidance maneuvre earlier and at a greater distance from an obstacle than when moving slowly.

Recently, we found that the responses of bee LWCs to visual stimuli as experienced during navigation flights in the vicinity of an invisible goal also strongly depend on the spatial layout of the environment. The spatial landmark constellation that guides the bees to their goal leads to a characteristic time-dependent response profile in LWCs during the intersaccadic intervals of navigation flights (Mertes et al. in preparation).

The responses of LWCs of flies and bees do not only depend on the retinal velocities, but are also sensitive to pattern properties (Figure 7; see above). Although the pattern-dependent modulations in the neural responses have been conventionally viewed as detrimental to the velocity signal, they may reflect functionally relevant information about the environment (Meyer et al., 2011; Hennig and Egelhaaf, 2012). This may be the case especially during intersaccadic translatory movements: since the retinal velocity scales with distance, an object nearby will lead to larger intersaccadic depolarization than a more distant one. Assuming that objects nearby are especially functionally relevant, object detection via optic flow automatically weighs objects according to their distance and, thus, their functional relevance. In other words, cluttered spatial scenery is segmented in this way, without much computational expenditure, into nearby and distant objects.

The amplitude of pattern-induced neural responses depends to a large extent on the size of the neuron's receptive field. Large receptive fields blur pattern-dependent response fluctuations and, thus, improve the quality of velocity signals (Figure 7). However, they do this at the expense of how well the signals can be localized. Hence, if motion signals originating from an object need to be localized by a neuron in the visual field, its receptive field should be sufficiently small; then, however, velocity coding is only poor and the signal provides local pattern information (Meyer et al., 2011). Hence, a neuron that is to encode spatial information on the basis of optic flow elicited during translatory self-motion should possess a receptive field that matches the size of the behaviorally relevant objects or textures. Sensitivity to objects may be further augmented by inhibitory spatial interactions, as is characteristic of blowfly FD cells (Hennig and Egelhaaf, 2012), and also by adaptive mechanisms (Liang et al., 2008, 2011). The enhanced sensitivity to objects in FD cells results from non-linearities in the synaptic interactions between an inhibitory neuron and the FD cell, on the one hand (Egelhaaf, 1985c; Hennig et al., 2008), and from the excitatory receptive field of the FD cell being smaller than that of its inhibitory input, on the other hand (Figures 6B–D) (Egelhaaf, 1985b; Egelhaaf et al., 1993; Krapp et al., 2001). In addition, the larger receptive field of the inhibitory LWC enhances the pattern-dependent response fluctuations in the FD cell (Hennig and Egelhaaf, 2012). Thus, the same mechanism which accounts for the FD cells being highly sensitive to objects defined by relative motion cues is also responsible for their sensitivity to objects which are defined by discontinuities in the textural properties of the environment.

It became evident in recent studies that the response properties of fly LWCs are affected by the behavioral state of the animal. Most prominently, the response amplitudes of LWCs increase if the animal is behaviorally active during the electrophysiological recording (Chiappe et al., 2010; Maimon et al., 2010; Rosner et al., 2010; Jung et al., 2011). This effect can be mimicked to some extent by application of the octopamine agonist CDM, which may induce an increase in overall spike rate and a slight shift in the velocity tuning (Longden and Krapp, 2009, 2010; Jung et al., 2011; de Haan et al., 2012; Rien et al., 2012). Octopamine has already been shown much earlier to increase the overall spike rate of LWCs in honeybees, although changes in velocity tuning have not been tested (Kloppenburg and Erber, 1995). These changes in LWC properties related to the behavioral state of the animal are unlikely to alter the conclusions about how environmental features are represented during intersaccadic LWC responses. High intersaccadic velocities, for instance, occur close to objects or the walls of the flight arena. A shift in velocity tuning toward higher velocities would reduce the likelihood of retinal velocities beyond the monotonic response range of the motion detection system and, thus, would improve the encoding of distance information.

We can conclude that LWCs of flies and bees provide information about the spatial layout and the pattern properties of the environment. This information is linked to the translational self-motion of the flying animal during intersaccadic intervals. As a consequence of the action–perception cycle and the distance dependence of translational optic flow, this spatial information is confined to the behaviorally relevant range of up to a few metres. Within this range, the animal has to take action, for instance, to avoid collisions with obstacles, to select a landing place or to employ environmental objects as landmarks in order to learn and/or find the location of a barely visible goal.

### Constraints set by a timescale of natural behavior

In classical behavioral paradigms using tethered flying insects, the experimenter-defined motion sequences usually stay constant on a timescale of several hundreds of milliseconds and even seconds. However, during unrestrained behavior, the retinal motion patterns continually change. As a consequence of the typical saccadic flight and gaze strategy of insects (see above), optic flow dynamics during natural locomotion also deviate considerably from dynamic stimuli (e.g., white-noise velocity fluctuations) that are often employed in characterizing LWCs. In the context of spatial vision, the intersaccadic intervals are of particular interest. Although they take up, on the whole, more than 80% of the entire flight time, they may be as short as 30 ms.

Why is the duration of intersaccadic intervals and, thus, the timescale on which information about the environment needs to be processed an issue at all? On the one hand, neurons are relatively unreliable computing devices and, on the other hand, the spatial behavior of flying insects takes place on a comparatively rapid timescale. The problem of reliability is particularly daunting, as there is not much redundancy at the output level of the insect visual system which would allow for the pooling of information across equivalent neurons.

When the same stimulus is presented repeatedly to a neuron, the responses may vary a lot between trials. Neuronal activity fluctuates continually even during constant velocity motion (reviews: Pelli, 1991; de Ruyter van Steveninck and Bialek, 1995; Warzecha and Egelhaaf, 2001). On the basis of individual response traces, it is not easily possible to discern stimulus-driven activity changes from those that are due to sources not associated with the stimulus (“noise”). The origin of various potential noise sources in the visual motion pathway and the consequences of the unreliable nature of neural signals have been analysed in flies (e.g., de Ruyter van Steveninck and Bialek, 1995; de Ruyter van Steveninck et al., 1997; Warzecha and Egelhaaf, 1999; Warzecha et al., 2000; Egelhaaf et al., 2001; Lewen et al., 2001; Borst, 2003; Grewe et al., 2003, 2007; Nemenman et al., 2008). These aspects, as well as the impact of neuronal noise on the precision with which motion information can be encoded, have been controversially discussed (Haag and Borst, 1997, 1998; Warzecha and Egelhaaf, 1997; Warzecha et al., 1998, 2000, 2003; Brenner et al., 2000b; Fairhall et al., 2001; Kalb, 2006). One aspect appears to be especially relevant in the context of computing spatial information: given that neuronal responses are noisy, it will take some time to infer reliably behaviorally relevant environmental information from neuronal activity. Bayesian analysis of noisy intersaccadic responses of individual fly LWCs and populations of LWCs reveals that sufficiently reliable information about translatory self-motion and, thus, about spatial parameters of the environment can be decoded already on a timescale of little more than 5 ms and, thus, on a time-scale of even the shortest intersaccadic intervals (Karmeier et al., 2005). Since the neural responses in this analysis were integrated over time, the intersaccadic responses decoded on this basis do not allow for resolving temporal response fluctuations that may arise from pattern properties during an intersaccadic interval. How much the neural responses fluctuate in a pattern-dependent way on a timescale of intersaccades needs to be investigated by scrutinizing individual responses to translations in natural surroundings.

## Conclusions

Despite their small brains with less than a million neurons and a spatial resolution of their eyes much smaller than any useful technical camera system, insects such as flies or bees are able to solve complex spatial tasks, such as avoiding collisions with obstacles, landing on objects or even finding hardly visible goals on the basis of spatial landmark information. Insects outperform man-made autonomous flying systems in these tasks especially if resource efficiency with respect to computational expenditure and energy consumption are conceived as a benchmark. Moreover, insects accomplish this at flight velocities that imply rapid time-varying retinal image flow. The processing of rapid retinal image flow represents great challenges for the neuronal machinery, given the limited reliability of neurons as computing devices. Obviously, as a consequence of millions of years of evolution, insect nervous systems have become well adapted to successfully cope with these computational challenges and to solve those computational tasks that are relevant for the success of the species efficiently and parsimoniously.

One means to accomplish their extraordinary performance is that flies and bees actively shape the image flow on their eyes by their characteristic flight behavior. Neural processing of spatial and textural information about the environment is greatly facilitated by largely segregating the rotational from the translational optic flow through a saccadic flight and gaze strategy. It is suggested that tuning the neural networks of motion computation to the specific spatiotemporal properties of the actively shaped optic flow patterns enables the nervous system to solve apparently complex spatial vision tasks more efficiently and parsimoniously than might be possible without such an active vision strategy. Only by taking into account the characteristics of the retinal image flow that is generated under natural closed-loop conditions did it become clear that the classical interpretations of the functional significance of neurons sensitive to optic flow need to be at least modified and extended: these neurons not only reflect information about the animals' self-motion, but also—through the image flow generated during intersaccadic translational movements—about the outside world. Accordingly, these neurons may be regarded as sensors for environmental information that, as a consequence of the distance dependence of translational optic flow, weigh in computationally inexpensive ways environmental information according to its presumptive significance for spatial vision.

Hence, we can conclude from the experimental work on the spatial behavior of insects and the underlying neural mechanisms, in combination with model simulations, that biological systems such as flies or bees derive part of their power as autonomous systems from scrutinizing their environment during the execution of sets of carefully selected motor routines, instead of just passively gathering information about the world. These animal–environment interactions lead to adaptive behavior in environments of a wide range of complexity. Model simulations and robotic implementations reveal that the smart biological mechanisms of motion computation and flight control might be helpful when designing micro air vehicles that may carry an on-board processor of only relatively small size and weight (Floreano et al., 2009).

### Conflict of interest statement

The authors declare that the research was conducted in the absence of any commercial or financial relationships that could be construed as a potential conflict of interest.

## References

[B1] Aptekar J. W. Shoemaker P. A. Frye M. A. (2012). Figure tracking by flies is supported by parallel visual streams. Curr. Biol. 22, 482–487 10.1016/j.cub.2012.01.044 22386313

[B2] Baird E. Kornfeldt T. Dacke M. (2010). Minimum viewing angle for visually guided ground speed control in bumblebees. J. Exp. Biol. 213, 1625–1632 10.1242/jeb.038802 20435812

[B3] Baird E. Srinivasan M. V. Zhang S. Cowling A. (2005). Visual control of flight speed in honeybees. J. Exp. Biol. 208, 3895–3905 10.1242/jeb.01818 16215217

[B4] Baird E. Srinivasan M. V. Zhang S. Lamont R. Cowling A. (2006). Visual control of flight speed and height in the honeybee, in From Animals to Animats 9, eds Nolfi S. Baldassare G. Calabretta R. Hallam J. Marocco D. Miglino O. (Berlin, Heidelberg: Springer; Lecture Notes in Computer Science), 40–51

[B5] Barnett P. D. Nordström K. O'Carroll D. C. (2007). Retinotopic organization of small-field-target-detecting neurons in the insect visual system. Curr. Biol. 17, 569–578 10.1016/j.cub.2007.02.039 17363248

[B6] Beersma D. G. M. Stavenga D. G. Kuiper J. W. (1977). Retinal lattice, visual field and binocularities in flies. J. Comp. Physiol. 119, 207–220

[B7] Bender J. A. Dickinson M. H. (2006a). A comparison of visual and haltere-mediated feedback in the control of body saccades in *Drosophila melanogaster*. J. Exp. Biol. 209, 4597–4606 10.1242/jeb.02583 17114395

[B8] Bender J. A. Dickinson M. H. (2006b). Visual stimulation of saccades in magnetically tethered Drosophila. J. Exp. Biol. 209, 3170–3182 10.1242/jeb.02369 16888065

[B9] Betsch B. Y. Einhäuser W. Körding K. P. König P. (2004). The world from a cat's perspective – statistics of natural videos. Biol. Cybern. 90, 41–50 10.1007/s00422-003-0434-6 14762723

[B10] Boeddeker N. Dittmar L. Stürzl W. Egelhaaf M. (2010). The fine structure of honeybee head and body yaw movements in a homing task. Proc. Biol. Sci. 277, 1899–1906 10.1098/rspb.2009.2326 20147329 PMC2871881

[B11] Boeddeker N. Hemmi J. M. (2010). Visual gaze control during peering flight manoeuvres in honeybees. Proc. Biol. Sci. 277, 1209–1217 10.1098/rspb.2009.1928 20007175 PMC2842814

[B12] Boeddeker N. Lindemann J. P. Egelhaaf M. Zeil J. (2005). Responses of blowfly motion-sensitive neurons to reconstructed optic flow along outdoor flight paths. J. Comp. Physiol. A 25, 1143–1155 10.1007/s00359-005-0038-9 16133502

[B13] Borst A. (2003). Noise, not stimulus entropy, determines neural information rate. J. Comput. Neurosci. 14, 23–31 12435922 10.1023/a:1021172200868

[B14] Borst A. (2009). Drosophila's view on insect vision. Curr. Biol. 19, R36–R47 10.1016/j.cub.2008.11.001 19138592

[B15] Borst A. Egelhaaf M. (1989). Principles of visual motion detection. Trends Neurosci. 12, 297–306 2475948 10.1016/0166-2236(89)90010-6

[B16] Borst A. Egelhaaf M. Haag J. (1995). Mechanisms of dendritic integration underlying gain control in fly motion-sensitive interneurons. J. Comput. Neurosci. 2, 5–18 8521280 10.1007/BF00962705

[B17] Borst A. Haag J. (2002). Neural networks in the cockpit of the fly. J. Comp. Physiol. A Neuroethol. Sens. Neural Behav. Physiol. 188, 419–437 10.1007/s00359-002-0316-8 12122462

[B18] Borst A. Haag J. Reiff D. F. (2010). Fly motion vision. Annu. Rev. Neurosci. 33, 49–70 10.1146/annurev-neuro-060909-153155 20225934

[B19] Borst A. Reisenman C. Haag J. (2003). Adaptation to response transients in fly motion vision: II. Model studies. Vision Res. 43, 1309–1322 10.1016/S0042-6989(03)00092-0 12726836

[B20] Borst A. Weber F. (2011). Neural action fields for optic flow based navigation: a simulation study of the fly lobula plate network. PLoS ONE 6:e16303 10.1371/journal.pone.0016303 21305019 PMC3031557

[B21] Braun E. Dittmar L. Boeddeker N. Egelhaaf M. (2012). Prototypical components of honeybee homing flight behaviour depend on the visual appearance of objects surrounding the goal. Front. Behav. Neurosci. 6:1 10.3389/fnbeh.2012.00001 22279431 PMC3260448

[B22] Braun E. Geurten B. Egelhaaf M. (2010). Identifying prototypical components in behaviour using clustering algorithms. PLoS ONE 5:e9361 10.1371/journal.pone.0009361 20179763 PMC2825265

[B23] Brenner N. Bialek W. de Ruyter van Steveninck R. (2000a). Adaptive rescaling maximizes information transmission. Neuron 26, 695–702 10.1016/S0896-6273(00)81205-2 10896164

[B24] Brenner N. Strong S. P. Koberle R. Bialek W. de Ruyter van Steveninck R. (2000b). Synergy in a neural code. Neural Comput. 12, 1531–1552 10935917 10.1162/089976600300015259

[B25] Brinkworth R. S. A. O'Carroll D. C. Graham L. J. (2009). Robust models for optic flow coding in natural scenes inspired by insect biology. PLoS Comput. Biol. 5:e1000555 10.1371/journal.pcbi.1000555 19893631 PMC2766641

[B26] Budick S. A. Dickinson M. H. (2006). Free-flight responses of *Drosophila melanogaster* to attractive odors. J. Exp. Biol. 209, 3001–3017 10.1242/jeb.02305 16857884

[B27] Budick S. A. Reiser M. B. Dickinson M. H. (2007). The role of visual and mechanosensory cues in structuring forward flight in *Drosophila melanogaster*. J. Exp. Biol. 210, 4092–4103 10.1242/jeb.006502 18025010

[B28] Chiappe M. E. Seelig J. D. Reiser M. B. Jayaraman V. (2010). Walking modulates speed sensitivity in *Drosophila* motion vision. Curr. Biol. 20, 1470–1475 10.1016/j.cub.2010.06.072 20655222 PMC4435946

[B29] Clark D. A. Bursztyn L. Horowitz M. A. Schnitzer M. J. Clandinin T. R. (2011). Defining the computational structure of the motion detector in Drosophila. Neuron 70, 1165–1177 10.1016/j.neuron.2011.05.023 21689602 PMC3121538

[B30] Clifford C. W. G. Ibbotson M. R. (2003). Fundamental mechanisms of visual motion detection: models, cells and functions. Prog. Neurobiol. 68, 409–437 10.1016/S0301-0082(02)00154-5 12576294

[B31] Collett T. S. (1978). Peering – a locust behavior pattern for obtaining motion parallax information. J. Exp. Biol. 76, 237–241

[B32] Collett T. S. Collett M. (2002). Memory use in insect visual navigation. Nat. Rev. Neurosci. 3, 542–552 10.1038/nrn872 12094210

[B33] Collett T. S. Graham P. Harris R. A. Hempel-De-Ibarra N. (2006). Navigational memories in ants and bees: memory retrieval when selecting and following routes. Adv. Study Behav. 36, 123–172

[B34] Collett T. S. Harkness L. I. K. (1982). Depth vision in animals, in Analysis of Visual Behaviour, eds Ingle D. J. Goodale M. A. Mansfield R. J. W. (Cambridge, MA; London England: The MIT Press), 111–176

[B35] Collett T. S. Land M. F. (1975). Visual control of flight behaviour in the hoverfly *Syritta pipiens* L. J. Comp. Physiol. 99, 1–66

[B36] Collett T. S. Paterson C. J. (1991). Relative motion parallax and target localization in the locust, *Schistocerca gregaria*. J. Comp. Physiol. A 169, 615–621

[B37] Dahmen H. J. Franz M. O. Krapp H. G. (2000). Extracting ego-motion from optic flow: limits of accuracy and neuronal filters, in Computational, Neural and Ecological Constraints of Visual Motion Processing, eds Zanker J. M. Zeil J. (Berlin, Heidelberg, New York: Springer), 143–168

[B38] David C. T. (1979). Optomotor control of speed and height by free-flying Drosophila. J. Exp. Biol. 82, 389–392 11799695 10.1242/jeb.82.1.389

[B39] David C. T. (1982). Compensation for height in the control of groundspeed by Drosophila in a new, ‘barber's pole’ wind tunnel. J. Comp. Physiol. 147, 485–493

[B40] Davies M. N. O. Green P. R. (1988). Head-bobbing during walking, running and flying: relative motion perception in the pigeon. J. Exp. Biol. 138, 71–91

[B41] de Haan R. Lee Y.-J. Nordström K. (2012). Octopaminergic modulation of contrast sensitivity. Front. Integr. Neurosci. 6:55 10.3389/fnint.2012.00055 22876224 PMC3411070

[B42] de Ruyter van Steveninck R. Bialek W. (1995). Reliability and statistical efficiency of a blowfly movement-sensitive neuron. Philos. Trans. R. Soc. Lond. B Biol. Sci. 348, 321–340

[B43] de Ruyter van Steveninck R. Lewen G. D. Strong S. P. Koberle R. Bialek W. (1997). Reproducibility and variability in neural spike trains. Science 275, 1805–1808 10.1126/science.275.5307.1805 9065407

[B44] DeVoe R. D. Kaiser W. Ohm J. Stone L. S. (1982). Horizontal movement detectors of honeybees. Directionally-selective visual neurons in the lobula and brain. J. Comp. Physiol. 147, 155–170

[B45] Dittmar L. Egelhaaf M. Stürzl W. Boeddeker N. (2011). The behavioural relevance of landmark texture for honeybee homing. Front. Behav. Neurosci. 5:20 10.3389/fnbeh.2011.00020 21541258 PMC3083717

[B46] Dittmar L. Stürzl W. Baird E. Boeddeker N. Egelhaaf M. (2010). Goal seeking in honeybees: matching of optic flow snapshots. J. Exp. Biol. 213, 2913–2923 10.1242/jeb.043737 20709919

[B47] Dong D. W. Attick J. J. (1995). Statistics of natural time-varying images. Network 6, 345–358

[B48] Dror R. O. O'Carroll D. C. Laughlin S. B. (2001). Accuracy of velocity estimation by Reichardt correlators. J. Opt. Soc. Am. A Opt. Image Sci. Vis. 18, 241–252 10.1364/JOSAA.18.000241 11205969

[B49] Duistermars B. J. Reiser M. B. Zhu Y. Frye M. A. (2007). Dynamic properties of large-field and small-field optomotor flight responses in Drosophila. J. Comp. Physiol. A Neuroethol. Sens. Neural Behav. Physiol. 193, 787–799 10.1007/s00359-007-0233-y 17551735

[B50] Dyhr J. P. Higgins C. M. (2010). The spatial frequency tuning of optic-flow-dependent behaviors in the bumblebee *Bombus impatiens*. J. Exp. Biol. 213, 1643–1650 10.1242/jeb.041426 20435814 PMC2861963

[B51] Eckert M. P. Buchsbaum G. (1993). Efficient coding of natural time varying images in the early visual system. Philos. Trans. R. Soc. Lond. B Biol. Sci. 339, 385–395 10.1098/rstb.1993.0038 8098870

[B52] Eckmeier D. Geurten B. R. H. Kress D. Mertes M. Kern R. Egelhaaf M. (2008). Gaze strategy in the free flying zebra finch (*Taeniopygia guttata*). PLoS ONE 3:e3956 10.1371/journal.pone.0003956 19107185 PMC2600564

[B53] Egelhaaf M. (1985a). On the neuronal basis of figure-ground discrimination by relative motion in the visual system of the fly. I. Behavioural constraints imposed on the neuronal network and the role of the optomotor system. Biol. Cybern. 52, 123–140

[B54] Egelhaaf M. (1985b). On the neuronal basis of figure-ground discrimination by relative motion in the visual system of the fly. II. Figure-Detection Cells, a new class of visual interneurones. Biol. Cybern. 52, 195–209

[B55] Egelhaaf M. (1985c). On the neuronal basis of figure-ground discrimination by relative motion in the visual system of the fly. III. Possible input circuitries and behavioural significance of the FD-Cells. Biol. Cybern. 52, 267–280

[B56] Egelhaaf M. (1987). Dynamic properties of two control systems underlying visually guided turning in house-flies. J. Comp. Physiol. A 161, 777–783

[B57] Egelhaaf M. (1989). Visual afferences to flight steering muscles controlling optomotor response of the fly. J. Comp. Physiol. A 165, 719–730 2810146 10.1007/BF00610871

[B58] Egelhaaf M. (2006). The neural computation of visual motion, in Invertebrate Vision, eds Warrant E. Nilsson D.-E. (Cambridge, UK: Cambridge University Press), 399–461

[B59] Egelhaaf M. Borst A. (1989). Transient and steady-state response properties of movement detectors. J. Opt. Soc. Am. A 6, 116–127 2921651 10.1364/josaa.6.000116

[B60] Egelhaaf M. Borst A. (1992). Are there separate on- and off-channels in fly motion vision? Vis. Neurosci. 8, 151–164 1558827 10.1017/s0952523800009317

[B61] Egelhaaf M. Borst A. (1993a). A look into the cockpit of the fly: visual orientation, algorithms, and identified neurons. J. Neurosci. 13, 4563–4574 8229185 10.1523/JNEUROSCI.13-11-04563.1993 PMC6576360

[B62] Egelhaaf M. Borst A. (1993b). Movement detection in arthropods, in Visual Motion and its Role in the Stabilization of Gaze, eds Miles F. A. Wallman J. (Amsterdam: Elsevier), 53–77 10.1007/s00429-008-0201-5

[B63] Egelhaaf M. Borst A. Reichardt W. (1989). Computational structure of a biological motion detection system as revealed by local detector analysis in the fly's nervous system. J. Opt. Soc. Am. A 6, 1070–1087 2760723 10.1364/josaa.6.001070

[B64] Egelhaaf M. Borst A. Warzecha A.-K. Flecks S. Wildemann A. (1993). Neural circuit tuning fly visual interneurons to motion of small objects. II. Input organization of inhibitory circuit elements by electrophysiological and optical recording techniques. J. Neurophysiol. 69, 340–351 8459271 10.1152/jn.1993.69.2.340

[B65] Egelhaaf M. Grewe J. Kern R. Warzecha A.-K. (2001). Outdoor performance of a motion-sensitive neuron in the blowfly. Vision Res. 41, 3627–3637 10.1016/S0042-6989(01)00220-6 11712978

[B66] Egelhaaf M. Kern R. Kurtz R. Krapp H. G. Kretzberg J. Warzecha A.-K. (2002). Neural encoding of behaviourally relevant motion information in the fly. Trends Neurosci. 25, 96–102 10.1016/S0166-2236(02)02063-5 11814562

[B67] Egelhaaf M. Reichardt W. (1987). Dynamic response properties of movement detectors: theoretical analysis and electrophysiological investigation in the visual system of the fly. Biol. Cybern. 56, 69–87 10.1016/j.neuroscience.2005.04.051 15975725

[B68] Egelhaaf M. Warzecha A.-K. (1998). Encoding of motion in real time by the fly visual system. Curr. Opin. Neurobiol. 9, 454–460 10448158 10.1016/s0959-4388(99)80068-3

[B69] Eichner H. Joesch M. Schnell B. Reiff D. F. Borst A. (2011). Internal structure of the fly elementary motion detector. Neuron 70, 1155–1164 10.1016/j.neuron.2011.03.028 21689601

[B70] Elyada Y. M. Haag J. Borst A. (2009). Different receptive fields in axons and dendrites underlie robust coding in motion-sensitive neurons. Nat. Neurosci. 12, 327–332 10.1038/nn.2269 19198603

[B71] Esch H. E. Zhang S. Srinivasan M. V. Tautz J. (2001). Honeybee dances communicate distances measured by optic flow. Nature 411, 581–583 10.1038/35079072 11385571

[B72] Fairhall A. L. Lewen G. D. Bialek W. de Ruyter van Steveninck R. (2001). Efficiency and ambiguity in an adaptive neural code. Nature 412, 787–792 10.1038/35090500 11518957

[B73] Farina W. M. Kramer D. Varjú D. (1995). The response of the hovering hawk moth *Macroglossum stellatarum* to translatory pattern motion. J. Comp. Physiol. 176, 551–562

[B74] Farina W. M. Varjú D. Zhou Y. (1994). The regulation of distance to dummy flowers during hovering flight in the hawk moth *Macroglossum stellatarum*. J. Comp. Physiol. 174, 239–247

[B75] Farrow K. Haag J. Borst A. (2003). Input organization of multifunctional motion-sensitive neurons in the blowfly. J. Neurosci. 29, 9805–9811 14586008 10.1523/JNEUROSCI.23-30-09805.2003 PMC6740885

[B76] Farrow K. Haag J. Borst A. (2006). Nonlinear, binocular interactions underlying flow field selectivity of a motion-sensitive neuron. Nat. Neurosci. 9, 1312–1320 10.1038/nn1769 16964250

[B77] Floreano D. Zufferey J.-C. Srinivasan M. V. Ellington C. (2009). Flying Insects and Robots. Heidelberg, Dordrecht, London, New York: Springer

[B78] Foucaud J. Burns J. G. Mery F. Zars T. (2010). Use of spatial information and search strategies in a water maze analog in *Drosophila melanogaster*. PLoS ONE 5:e15231 10.1371/journal.pone.0015231 21151940 PMC2997081

[B79] Fry S. N. Rohrseitz N. Straw A. D. Dickinson M. H. (2009). Visual control of flight speed in *Drosophila melanogaster*. J. Exp. Biol. 212, 1120–1130 10.1242/jeb.020768 19329746

[B80] Frye M. A. Dickinson M. H. (2007). Visual edge orientation shapes free-flight behavior in Drosophila. Fly 1, 153–154 18820449 10.4161/fly.4563

[B81] Geisler W. S. (2008). Visual perception and the statistical properties of natural scenes. Annu. Rev. Psychol. 59, 10.1–10.26 10.1146/annurev.psych.58.110405.085632 17705683

[B82] Geurten B. R. H. Kern R. Braun E. Egelhaaf M. (2010). A syntax of hoverfly flight prototypes. J. Exp. Biol. 213, 2461–2475 10.1242/jeb.036079 20581276

[B83] Geurten B. R. H. Kern R. Egelhaaf M. (2012). Species-specific flight styles of flies are reflected in the response dynamics of a homolog motion-sensitive neuron. Front. Integr. Neurosci. 6:11 10.3389/fnint.2012.00011 22485089 PMC3307035

[B84] Geurten B. R. H. Nordström K. Sprayberry J. D. H. Bolzon D. M. O'Carroll D. C. (2007). Neural mechanisms underlying target detection in a dragonfly centrifugal neuron. J. Exp. Biol. 219, 3277–3284 10.1242/jeb.008425 17766305

[B85] Gilbert C. Strausfeld N. J. (1991). The functional organization of male-specific visual neurons in flies. J. Comp. Physiol. A 169, 395–411 1723431 10.1007/BF00197653

[B86] Götz K. G. (1965). Die optischen Übertragungseigenschaften der Komplexaugen von Drosophila. Kybernetik 2, 215–221 5839008 10.1007/BF00306417

[B87] Götz K. G. (1987). Course-control, metabolism and wing interference during ultralong tethered flight in *Drosophila melanogaster*. J. Exp. Biol. 128, 35–46

[B88] Grewe J. Kretzberg J. Warzecha A.-K. Egelhaaf M. (2003). Impact of photon-noise on the reliability of a motion-sensitive neuron in the fly's visual system. J. Neurosci. 23, 10776–10783 14645469 10.1523/JNEUROSCI.23-34-10776.2003 PMC6740987

[B89] Grewe J. Weckström M. Egelhaaf M. Warzecha A.-K. (2007). Information and discriminability as measures of reliability of sensory coding. PLoS ONE 2:e1328 10.1371/journal.pone.0001328 18091998 PMC2121128

[B90] Gronenberg W. Milde J. J. Strausfeld N. J. (1995). Oculomotor control in Calliphorid flies: organization of descending neurons to neck motor neurons responding to visual stimuli. J. Comp. Neurol. 361, 267–284 10.1002/cne.903610206 8543662

[B91] Gronenberg W. Strausfeld N. J. (1990). Descending neurons supplying the neck and flight motor of Diptera: physiological and anatomical characteristics. J. Comp. Neurol. 302, 973–991 10.1002/cne.903020420 1707070

[B92] Haag J. Borst A. (1997). Encoding of visual motion information and reliability in spiking and graded potential neurons. J. Neurosci. 17, 4809–4819 9169539 10.1523/JNEUROSCI.17-12-04809.1997 PMC6573340

[B93] Haag J. Borst A. (1998). Active membrane properties and signal encoding in graded potential neurons. J. Neurosci. 18, 7972–7986 9742164 10.1523/JNEUROSCI.18-19-07972.1998 PMC6793010

[B94] Hardie R. C. (1985). Functional organization of the fly retina, in Progress in Sensory Physiology 5, eds Autrum H. Ottoson D. Perl E. R. Schmidt R. F. Shimazu H. Willis W. D. (Berlin, Heidelberg, New York, Tokyo: Springer), 1–79

[B95] Harris R. A. O'Carroll D. C. Laughlin S. B. (2000). Contrast gain reduction in fly motion adaptation. Neuron 28, 595–606 10.1016/S0896-6273(00)00136-7 11144367

[B96] Hausen K. (1982). Motion sensitive interneurons in the optomotor system of the fly. II. The horizontal cells: receptive field organization and response characteristics. Biol. Cybern. 46, 67–79

[B97] Hausen K. Egelhaaf M. (1989). Neural mechanisms of visual course control in insects, in Facets of Vision, eds Stavenga D. G. Hardie R. C. (Berlin, Heidelberg: Springer), 391–424

[B98] Heisenberg M. Wolf R. (1979). On the fine structure of yaw torque in visual flight orientation of *Drosophila melanogaster*. J. Comp. Physiol. 130, 113–130

[B99] Heitwerth J. Kern R. van Hateren J. H. Egelhaaf M. (2005). Motion adaptation leads to parsimonious encoding of natural optic flow by blowfly motion vision system. J. Neurophysiol. 94, 1761–1769 10.1152/jn.00308.2005 15917319

[B100] Hengstenberg R. (1993). Multisensory control in insect oculomotor systems, in Visual Motion and its Role in the Stabilization of Gaze, Bd 1, eds Miles F. A. Wallman J. (Amsteram, London, New York, Tokyio: Elsevier), 285–298 8420553

[B101] Hennig P. Egelhaaf M. (2012). Neuronal encoding of object and distance information: a model simulation study on naturalistic optic flow processing. Front. Neural Circuits 6:14 10.3389/fncir.2012.00014 22461769 PMC3309705

[B102] Hennig P. Kern R. Egelhaaf M. (2011). Binocular integration of visual information: a model study on naturalistic optic flow processing. Front. Neural Circuits 5:4 10.3389/fncir.2011.00004 21519385 PMC3078557

[B103] Hennig P. Möller R. Egelhaaf M. (2008). Distributed dendritic processing facilitates object detection: a computational analysis on the visual system of the fly. PLoS ONE 3:e3092 10.1371/journal.pone.0003092 18769475 PMC2517649

[B104] Horstmann W. Egelhaaf M. Warzecha A.-K. (2000). Synaptic interactions increase optic flow specificity. Eur. J. Neurosci. 12, 2157–2165 10.1046/j.1460-9568.2000.00094.x 10886355

[B105] Huston S. J. Krapp H. G. (2008). Visuomotor transformation in the fly gaze stabilization system. PLoS Biol. 6:e173 10.1371/journal.pbio.0060173 18651791 PMC2475543

[B106] Huston S. J. Krapp H. G. (2009). Nonlinear integration of visual and haltere inputs in fly neck motor neurons. J. Neurosci. 29, 13097–13105 10.1523/JNEUROSCI.2915-09.2009 19846697 PMC6665201

[B107] Ibbotson M. R. (1991). Wide-field motion-sensitive neurons tuned to horizontal movement in the honeybee, *Apis mellifera*. J. Comp. Physiol. A 168, 91–102

[B108] Joesch M. Plett J. Borst A. Reiff D. F. (2008). Response properties of motion-sensitive visual interneurons in the lobula plate of *Drosophila melanogaster*. Curr. Biol. 18, 368–374 10.1016/j.cub.2008.02.022 18328703

[B109] Joesch M. Schnell B. Raghu S. V. Reiff D. F. Borst A. (2010). ON and OFF pathways in Drosophila motion vision. Nature 468, 300–304 10.1038/nature09545 21068841

[B110] Jung S. N. Borst A. Haag J. (2011). Flight activity alters velocity tuning of fly motion-sensitive neurons. J. Neurosci. 31, 9231–9237 10.1523/JNEUROSCI.1138-11.2011 21697373 PMC6623490

[B111] Juusola M. (2003). The rate of information transfer of naturalistic stimulation by graded potentials. J. Gen. Physiol. 122, 191–206 10.1085/jgp.200308824 12860926 PMC2229540

[B112] Juusola M. French A. S. Uusitalo R. O. Weckström M. (1996). Information processing by graded-potential transmission through tonically active synapses. Trends Neurosci. 19, 292–297 10.1016/S0166-2236(96)10028-X 8799975

[B113] Juusola M. Kouvalainen E. Järvilehto M. Weckström M. (1994). Contrast gain, signal-to-noise ratio and linearity in light-adapted blowfly photoreceptors. J. Gen. Physiol. 104, 593–621 10.1085/jgp.104.3.593 7807062 PMC2229225

[B114] Juusola M. Uusitalo R. O. Weckström M. (1995). Transfer of graded potentials at the photoreceptor-interneuron synapse. J. Gen. Physiol. 103, 117–148 10.1085/jgp.105.1.117 7537323 PMC2216927

[B115] Kalb J. (2006). Robust integration of motion information in the fly visual system revealed by single cell photoablation. J. Neurosci. 26, 7898–7906 10.1523/JNEUROSCI.1327-06.2006 16870735 PMC6674221

[B116] Kalb J. Egelhaaf M. Kurtz R. (2008). Adaptation changes directional sensitivity in a visual motion-sensitive neuron of the fly. Vision Res. 48, 1735–1742 10.1016/j.visres.2008.05.004 18556040

[B117] Karmeier K. Krapp H. G. Egelhaaf M. (2003). Robustness of the tuning of fly visual interneurons to rotatory optic flow. J. Neurophysiol. 90, 1626–1634 10.1152/jn.00234.2003 12736239

[B118] Karmeier K. Krapp H. G. Egelhaaf M. (2005). Population coding of self-motion: applying Bayesian analysis to a population of visual interneurons in the fly. J. Neurophysiol. 94, 2182–2194 10.1152/jn.00278.2005 15901759

[B119] Karmeier K. van Hateren J. H. Kern R. Egelhaaf M. (2006). Encoding of naturalistic optic flow by a population of blowfly motion sensitive neurons. J. Neurophysiol. 96, 1602–1614 10.1152/jn.00023.2006 16687623

[B120] Katsov A. Y. Clandinin T. R. (2008). Motion processing streams in Drosophila are behaviorally specialized. Neuron 59, 322–335 10.1016/j.neuron.2008.05.022 18667159 PMC3391501

[B121] Kern R. Boeddeker N. Dittmar L. Egelhaaf M. (2012). Blowfly flight characteristics are shaped by environmental features and controlled by optic flow information. J. Exp. Biol. 215, 2501–2514 10.1242/jeb.061713 22723490

[B122] Kern R. Egelhaaf M. Srinivasan M. V. (1997). Edge detection by landing honeybees: behavioural analysis and model simulations of the underlying mechanism. Vision Res. 37, 2103–2117 10.1016/S0042-6989(97)00013-8 9327058

[B123] Kern R. Lutterklas M. Egelhaaf M. (2000). Neural representation of optic flow experienced by unilaterally blinded flies on their mean walking trajectories. J. Comp. Physiol. A 186, 467–479 10.1007/s003590050445 10879949

[B124] Kern R. van Hateren J. H. Egelhaaf M. (2006). Representation of behaviourally relevant information by blowfly motion-sensitive visual interneurons requires precise compensatory head movements. J. Exp. Biol. 209, 1251–1260 10.1242/jeb.02127 16547297

[B125] Kern R. van Hateren J. H. Michaelis C. Lindemann J. P. Egelhaaf M. (2005). Function of a fly motion-sensitive neuron matches eye movements during free flight. PLoS Biol. 3:e171 10.1371/journal.pbio.0030171 15884977 PMC1110907

[B126] Kern R. Varjú D. (1998). Visual position stabilization in the hummingbird hawk moth, Macroglossum stellatarum L.: I. Behavioural analysis. J. Comp. Physiol. A 182, 225–237 10.1007/s003590050173 9463920

[B127] Kimmerle B. Egelhaaf M. (2000a). Detection of object motion by a fly neuron during simulated translatory flight. J. Comp. Physiol. A 186, 21–31 10.1007/s003590050003 10659039

[B128] Kimmerle B. Egelhaaf M. (2000b). Performance of fly visual interneurons during object fixation. J. Neurosci. 20, 6256–6266 10934276 10.1523/JNEUROSCI.20-16-06256.2000 PMC6772600

[B129] Kimmerle B. Eikermann J. Egelhaaf M. (2000). Object fixation by the blowfly during tethered flight in a simulated three-dimensional environment. J. Exp. Biol. 203, 1723–1732 10804162 10.1242/jeb.203.11.1723

[B130] Kimmerle B. Srinivasan M. V. Egelhaaf M. (1996). Object detection by relative motion in freely flying flies. Naturwiss 83, 380–381

[B131] Kimmerle B. Warzecha A.-K. Egelhaaf M. (1997). Object detection in the fly during simulated translatory flight. J. Comp. Physiol. A 181, 247–255 10.1084/jem.181.1.247 7528770 PMC2191817

[B132] Kirschfeld K. (1972). The visual system of Musca: studies on optics, structure and function, in Information Processing in the Visual System of Arthropods, ed Wehner R. (Berlin, Heidelberg, New York: Springer), 61–74

[B133] Kloppenburg P. Erber J. (1995). The modulatory effects of serotonin and octopamine in the visual-system of the honey-bee (*Apis mellifera* L).2. Electrophysiological analysis of motion-sensitive neurons in the lobula. J. Comp. Physiol. A 176, 119–129

[B134] Koenderink J. J. (1986). Optic flow. Vision Res. 26, 161–180 10.1016/0042-6989(86)90078-7 3716209

[B135] Kral K. Poteser M. (1997). Motion parallax as a source of distance information in locusts and mantids. J. Insect Behav. 10, 145–163

[B136] Krapp H. G. (2000). Neuronal matched filters for optic flow processing in flying insects, in Neuronal Processing of Optic Flow, International Review of Neurobiology, Vol. 44, ed Lappe M. (San Diego, CA: Academic Press), 93–120 10.1016/s0074-7742(08)60739-4 10605643

[B137] Krapp H. G. Hengstenberg B. Hengstenberg R. (1998). Dendritic structure and receptive-field organization of optic flow processing interneurons in the fly. J. Neurophysiol. 79, 1902–1917 9535957 10.1152/jn.1998.79.4.1902

[B138] Krapp H. G. Hengstenberg R. (1996). Estimation of self-motion by optic flow processing in single visual interneurons. Nature 384, 463–466 10.1038/384463a0 8945473

[B139] Krapp H. G. Hengstenberg R. Egelhaaf M. (2001). Binocular contribution to optic flow processing in the fly visual system. J. Neurophysiol. 85, 724–734 11160507 10.1152/jn.2001.85.2.724

[B140] Kurtz R. (2007). Direction-selective adaptation in fly visual motion-sensitive neurons is generated by an intrinsic conductance-based mechanism. Neuroscience 146, 573–583 10.1016/j.neuroscience.2007.01.058 17367948

[B141] Kurtz R. (2009). The many facets of adaptation in fly visual motion processing. Commun. Integr. Biol. 2, 1–3 10.4161/cib.2.1.7350 19704857 PMC2649291

[B142] Kurtz R. (2012). Adaptive encoding of motion information in the fly visual system, in Frontiers in Sensing, eds Barth F. Humphrey J. Srinivasan M. (Wien, New York: Springer), 115–128 10.1523/JNEUROSCI.1936-08.2008

[B143] Kurtz R. Egelhaaf M. Meyer H. G. Kern R. (2009). Adaptation accentuates responses of fly motion-sensitive visual neurons to sudden stimulus changes. Proc. Biol. Sci. 276, 3711–3719 10.1098/rspb.2009.0596 19656791 PMC2817298

[B144] Land M. F. Collett T. S. (1974). Chasing behaviour of houseflies (*Fannia canicularis*). A description and analysis. J. Comp. Physiol. 89, 331–357 10.1080/00071666808415728 5687052

[B145] Lappe M. (ed.). (2000). Neuronal Processing of Optic Flow. San Diego, CA: Academic Press (International Review of Neurobiology)

[B146] Laughlin S. B. (1994). Matching coding, circuits, cells, and molecules to signals: general principles of retinal design in the fly's eye. Prog. Ret. Eye Res. 13, 165–196

[B147] Lehrer M. Campan R. (2005). Generalization of convex shapes by bees: what are shapes made of? J. Exp. Biol. 208, 3233–3247 10.1242/jeb.01790 16109886

[B148] Lehrer M. Srinivasan M. V. Zhang S. W. Horridge G. A. (1988). Motion cues provide the bee's visual world with a third dimension. Nature 332, 356–357

[B149] Lewen G. D. Bialek W. de Ruyter van Steveninck R. (2001). Neural coding of naturalistic motion stimuli. Network 12, 317–329 11563532

[B150] Liang P. Heitwerth J. Kern R. Kurtz R. Egelhaaf M. (2012). Object representation and distance encoding in three-dimensional environments by a neural circuit in the visual system of the blowfly. J. Neurophysiol. 107, 3446–3457 10.1152/jn.00530.2011 22423002

[B151] Liang P. Kern R. Egelhaaf M. (2008). Motion adaptation enhances object-induced neural activity in three-dimensional virtual environment. J. Neurosci. 28, 1–6 10.1523/JNEUROSCI.0203-08.2008 18971474 PMC6671490

[B152] Liang P. Kern R. Kurtz R. Egelhaaf M. (2011). Impact of visual motion adaptation on neural responses to objects and its dependence on the temporal characteristics of optic flow. J. Neurophysiol. 105, 1825–1834 10.1152/jn.00359.2010 21307322

[B153] Lindemann J. P. Kern R. Michaelis C. Meyer P. van Hateren J. H. Egelhaaf M. (2003). FliMax, a novel stimulus device for panoramic and highspeed presentation of behaviourally generated optic flow. Vision Res. 43, 779–791 10.1016/S0042-6989(03)00039-7 12639604

[B154] Lindemann J. P. Kern R. van Hateren J. H. Ritter H. Egelhaaf M. (2005). On the computations analysing natural optic flow: quantitative model analysis of the blowfly motion vision pathway. J. Neurosci. 25, 6435–6448 10.1523/JNEUROSCI.1132-05.2005 16000634 PMC6725274

[B155] Lindemann J. P. Weiss H. Möller R. Egelhaaf M. (2008). Saccadic flight strategy facilitates collision avoidance: closed-loop performance of a cyberfly. Biol. Cybern. 98, 213–227 10.1007/s00422-007-0205-x 18180948

[B156] Longden K. D. Krapp H. G. (2009). State-dependent performance of optic-flow processing interneurons. J. Neurophysiol. 102, 3606–3618 10.1152/jn.00395.2009 19812292

[B157] Longden K. D. Krapp H. G. (2010). Octopaminergic modulation of temporal frequency coding in an identified optic flow-processing interneuron. Front. Syst. Neurosci. 4:153 10.3389/fnsys.2010.00153 21152339 PMC2996258

[B158] Longuet-Higgins H. C. Prazdny K. (1980). The interpretation of a moving retinal image. Proc. R. Soc. Lond. B Biol. Sci. 208, 385–397 6106198 10.1098/rspb.1980.0057

[B159] Maddess T. Laughlin S. B. (1985). Adaptation of the motion-sensitive neuron H1 is generated locally and governed by contrast frequency. Proc. R. Soc. Lond. B Biol. Sci. 225, 251–275

[B160] Maimon G. Straw A. D. Dickinson M. H. (2008). A simple vision-based algorithm for decision making in flying Drosophila. Curr. Biol. 18, 464–470 10.1016/j.cub.2008.02.054 18342508

[B161] Maimon G. Straw A. D. Dickinson M. H. (2010). Active flight increases the gain of visual motion processing in *Drosophila*. Nat. Neurosci. 13, 393–399 10.1038/nn.2492 20154683

[B162] Meyer H. G. Lindemann J. P. Egelhaaf M. (2011). Pattern-dependent response modulations in motion-sensitive visual interneurons – A model study. PLoS ONE 6:e21488 10.1371/journal.pone.0021488 21760894 PMC3132178

[B163] Milde J. J. Gronenberg W. Strausfeld N. J. (1995). Oculomotor control in calliphorid flies: organization of descending neurons to neck motor neurons responding to visual stimuli. J. Comp. Neurol. 361, 267–284 10.1002/cne.903610206 8543662

[B164] Milde J. J. Seyan H. S. Strausfeld N. J. (1987). The neck motor system of the fly, *Calliphora erythrocephala*. II. Sensory organization. J. Comp. Physiol. A 160, 225–238

[B165] Mronz M. Lehmann F.-O. (2008). The free-flight response of Drosophila to motion of the visual environment. J. Exp. Biol. 211, 2026–2045 10.1242/jeb.008268 18552291

[B166] Necker R. (2007). Head-bobbing of walking birds. J. Comp. Physiol. A Neuroethol. Sens. Neural Behav. Physiol. 193, 1177–1183 10.1007/s00359-007-0281-3 17987297

[B167] Nemenman I. Lewen G. D. Bialek W. de Ruyter van Steveninck R. R. Friston K. J. (2008). Neural coding of natural stimuli: information at sub-millisecond resolution. PLoS Comput. Biol. 4:e1000025 10.1371/journal.pcbi.1000025 18369423 PMC2265477

[B168] Nordström K. (2012). Neural specializations for small target detection in insects. Curr. Opin. Neurobiol. 22, 272–278 10.1016/j.conb.2011.12.013 22244741

[B169] Nordström K. Barnett P. D. O'Carroll D. C. (2006). Insect detection of small targets moving in visual clutter. PLoS Biol. 4:e54 10.1371/journal.pbio.0040054 16448249 PMC1360098

[B170] Nordström K. O'Carroll D. C. (2006). Small object detection neurons in female hoverflies. Proc. Biol. Sci. 273, 1211–1216 10.1098/rspb.2005.3424 16720393 PMC1560283

[B171] O'Carroll D. C. Barnett P. D. Nordström K. (2011). Local and global responses of insect motion detectors to the spatial structure of natural scenes. J. Vis. 11, 1–17 Available online at: http://www.journalofvision.org/content/11/14/20 10.1167/11.14.20 22201615

[B172] Ofstad T. A. Zuker C. S. Reiser M. B. (2011). Visual place learning in *Drosophila melanogaster*. Nature 474, 204–207 10.1038/nature10131 21654803 PMC3169673

[B173] Olberg R. M. (1981). Object- and self-movement detectors in the ventral nerve cord of the dragonfly. J. Comp. Physiol. 141, 327–334

[B174] Olberg R. M. (1986). Identified target-selective visual interneurons descending from the dragonfly brain. J. Comp. Physiol. 159, 827–840

[B175] Olberg R. M. Worthington A. H. Fox J. L. Bessette C. E. Loosemore M. P. (2005). Prey size selection and distance estimation in foraging adult dragonflies. J. Comp. Physiol. A Neuroethol. Sens. Neural Behav. Physiol. 191, 791–797 10.1007/s00359-005-0002-8 16034603

[B176] Pelli D. G. (1991). Noise in the visual system may be early, in Computational Models of Visual Processing, eds Landy M. S. Movshon J. A. (Cambridge, MA: MIT-Press), 147–151

[B177] Petrowitz R. Dahmen H. J. Egelhaaf M. Krapp H. G. (2000). Arrangement of optical axes and the spatial resolution in the compound eye of the female blowfly *Calliphora*. J. Comp. Physiol. A 186, 737–746 10.1007/s003590000127 11016789

[B178] Pfaff M. Varjú D. (1991). Mechanisms of visual distance perception in the hawk moth *Macroglossum stellatarum*. Zool. Jahrb. Physiol. 95, 315–321

[B179] Portelli G. Ruffier F. Roubieu F. L. Franceschini N. Krapp H. G. (2011). Honeybees' speed depends on dorsal as well as lateral, ventral and frontal optic flows. PLoS ONE 6:e19486 10.1371/journal.pone.0019486 21589861 PMC3093387

[B180] Poteser M. Kral K. (1995). Visual distance discrimination between stationary targets in praying mantis: an index of the use of motion parallax. J. Exp. Biol. 198, 2127–2137 9320039 10.1242/jeb.198.10.2127

[B181] Prazdny K. (1980). Ego-motion and relative depth map from optical-flow. Biol. Cybern. 36, 87–102 7353067 10.1007/BF00361077

[B182] Redlick F. P. Jenkin M. Harris L. R. (2001). Humans can use optic flow to estimate distance of travel. Vision Res. 41, 213–219 10.1016/S0042-6989(00)00243-1 11163855

[B183] Reichardt W. (1961). Autocorrelation, a principle for the evaluation of sensory information by the central nervous system, in Sensory Communication, ed Rosenblith W. A. (New York, London: MIT Press and John Wiley and Sons), 303–317

[B184] Reichardt W. Poggio T. (1979). Figure-ground discrimination by relative movement in the visual system of the fly. Part I: experimental results. Biol. Cybern. 35, 81–100

[B185] Reichardt W. Poggio T. Hausen K. (1983). Figure-ground discrimination by relative movement in the visual system of the fly. Part II: towards the neural circuitry. Biol. Cybern. 46, 1–30

[B186] Reiff D. F. Plett J. Mank M. Griesbeck O. Borst A. (2010). Visualizing retinotopic half-wave rectified input to the motion detection circuitry of Drosophila. Nat. Neurosci. 13, 973–978 10.1038/nn.2595 20622873

[B187] Reiser M. B. Dickinson M. H. (2010). Drosophila fly straight by fixating objects in the face of expanding optic flow. J. Exp. Biol. 213, 1771–1781 10.1242/jeb.035147 20435828 PMC2861965

[B188] Rien D. Kern R. Kurtz R. (2012). Octopaminergic modulation of contrast gain adaptation in fly visual motion-sensitive neurons. Eur. J. Neurosci. 36, 3030–3039 10.1111/j.1460-9568.2012.08216.x 22775326

[B189] Rister J. Pauls D. Schnell B. Ting C. Y. Lee C. H. Sinakevitch I. (2007). Dissection of the peripheral motion channel in the visual system of *Drosophila melanogaster*. Neuron 56, 155–170 10.1016/j.neuron.2007.09.014 17920022

[B190] Ristroph L. Berman G. J. Bergou A. J. Wang Z. J. Cohen I. (2009). Automated hull reconstruction motion tracking (HRMT) applied to sideways maneuvers of free flying insects. J. Exp. Biol. 212, 1324–1335 10.1242/jeb.025502 19376953

[B191] Rogers B. J. (1993). Motion parallax and other dynamic cues for depth vision, in Visual motion and its role in the stabilization of gaze, eds Miles F. A. Wallman J. (Amsterdam: Elsevier), 119–137

[B192] Rosner R. Egelhaaf M. Grewe J. Warzecha A.-K. (2009). Variability of blowfly head optomotor responses. J. Exp. Biol. 212, 1170–1184 10.1242/jeb.027060 19329750

[B193] Rosner R. Egelhaaf M. Warzecha A.-K. (2010). Behavioural state affects motion-sensitive neurones in the fly visual system. J. Exp. Biol. 213, 331–338 10.1242/jeb.035386 20038668

[B194] Sandeman D. C. Markl H. (1980). Head movements in the flies (*Calliphora*) produced by deflexion of the halteres. J. Exp. Biol. 85, 43–60

[B195] Schilstra C. van Hateren J. H. (1999). Blowfly flight and optic flow. I. Thorax kinematics and flight dynamics. J. Exp. Biol. 202, 1481–1490 10229694 10.1242/jeb.202.11.1481

[B196] Schnell B. Raghu S. V. Nern A. Borst A. (2012). Columnar cells necessary for motion responses of wide-field visual interneurons in *Drosophila*. J. Comp. Physiol. A Neuroethol. Sens. Neural Behav. Physiol. 198, 389–395 10.1007/s00359-012-0716-3 22411431 PMC3332379

[B197] Schuster S. Strauss R. Götz K. G. (2002). Virtual-reality techniques resolve the visual cues used by fruit flies to evaluate object distances. Curr. Biol. 12, 1591–1594 10.1016/S0960-9822(02)01141-7 12372251

[B198] Seidl R. Kaiser W. (1981). Visual field size, binocular domain and the ommatidial array of the compound eyes in worker honey bees. J. Comp. Physiol. 143, 17–26

[B199] Sherman A. (2003). A comparison of visual and haltere-mediated equilibrium reflexes in the fruit fly *Drosophila melanogaster*. J. Exp. Biol. 206, 295–302 12477899 10.1242/jeb.00075

[B200] Sherman A. Dickinson M. H. (2003). A comparison of visual and haltere-mediated equilibrium reflexes in the fruit fly *Drosophila melanogaster*. J. Exp. Biol. 206, 295–302 10.1242/jeb.00075 12477899

[B201] Sherman A. Dickinson M. H. (2004). Summation of visual and mechanosensory feedback in Drosophila flight control. J. Exp. Biol. 207, 133–142 10.1242/jeb.00731 14638840

[B202] Simoncelli E. P. Olshausen B. A. (2001). Natural image statistics and neural representation. Annu. Rev. Neurosci. 24, 1193–1225 10.1146/annurev.neuro.24.1.1193 11520932

[B203] Single S. Borst A. (1998). Dendritic integration and its role in computing image velocity. Science 281, 1848–1850 10.1126/science.281.5384.1848 9743497

[B204] Single S. Haag J. Borst A. (1997). Dendritic computation of direction selectivity and gain control in visual interneurons. J. Neurosci. 17, 6023–6030 9236213 10.1523/JNEUROSCI.17-16-06023.1997 PMC6568333

[B205] Sobel E. C. (1990). The locust's use of motion parallax to measure distance. J. Comp. Physiol. A 167, 579–588 2074547 10.1007/BF00192653

[B206] Srinivasan M. Lehrer M. Wehner R. (1987). Bees perceive illusory colours induced by movement. Vision Res. 27, 1285–1289 3424676 10.1016/0042-6989(87)90205-7

[B207] Srinivasan M. V. Lehrer M. Horridge G. A. (1990). Visual figure-ground discrimination in the honeybee: the role of motion parallax at boundaries. Proc. R. Soc. Lond. B 238, 331–350

[B208] Srinivasan M. V. Lehrer M. Kirchner W. H. Zhang S. W. (1991). Range perception through apparent image speed in freely flying honeybees. Vis. Neurosci. 6, 519–535 2069903 10.1017/s095252380000136x

[B209] Srinivasan M. V. Zhang S. Altwein M. Tautz J. (2000). Honeybee navigation: nature and calibration of the “odometer”. Science 287, 851–853 10.1126/science.287.5454.851 10657298

[B210] Srinivasan M. V. Zhang S. W. Lehrer M. Collett T. S. (1996). Honeybee navigation en route to the goal: visual flight control and odometry. J. Exp. Biol. 199, 237–244 9317712 10.1242/jeb.199.1.237

[B211] Strausfeld N. J. Douglass J. K. Campbell H. R. Higgins C. M. (2006). Parallel processing in the optic lobes of flies and the occurrence of motion computing circuits, in Invertebrate Vision, eds Warrant E. Nilsson D.-E. (Cambridge, UK: Cambridge University Press), 349–398

[B212] Straw A. D. Lee S. Dickinson M. H. (2010). Visual control of altitude in flying drosophila. Curr. Biol. 20, 1550–1556 10.1016/j.cub.2010.07.025 20727759

[B213] Straw A. D. Rainsford T. O'Carroll D. C. (2008). Contrast sensitivity of insect motion detectors to natural images. J. Vis. 8, 1–9 Available online at: http://journalofvision.org/8/3/32/ 10.1167/8.3.32 18484838

[B214] Stürzl W. Zeil J. (2007). Depth, contrast and view-based homing in outdoor scenes. Biol. Cybern. 96, 519–531 10.1007/s00422-007-0147-3 17443340

[B215] Tammero L. F. Dickinson M. H. (2002a). Collision-avoidance and landing responses are mediated by separate pathways in the fruit fly, *Drosophila melanogaster*. J. Exp. Biol. 205, 2785–2798 12177144 10.1242/jeb.205.18.2785

[B216] Tammero L. F. Dickinson M. H. (2002b). The influence of visual landscape on the free flight behavior of the fruit fly *Drosophila melanogaster*. J. Exp. Biol. 205, 327–343 11854370 10.1242/jeb.205.3.327

[B217] Tammero L. F. Frye M. A. Dickinson M. H. (2004). Spatial organization of visuomotor reflexes in Drosophila. J. Exp. Biol. 207, 113–122 10.1242/jeb.00724 14638838

[B218] Taylor G. K. Krapp H. G. (2008). Sensory systems and flight stability: what do insects measure and why? in Advances in Insect Physiology (Amsterdam: Elsevier), 231–316

[B219] Trischler C. Boeddeker N. Egelhaaf M. (2007). Characterisation of a blowfly male-specific neuron using behaviourally generated visual stimuli. J. Comp. Physiol. A Neuroethol. Sens. Neural. Behav. Physiol. 193, 559–572 10.1007/s00359-007-0212-3 17333206

[B220] Trischler C. Kern R. Egelhaaf M. (2010). Chasing behavior and optomotor following in free-flying male blowflies: flight performance and interactions of the underlying control systems. Front. Behav. Neurosci. 4:20 10.3389/fnbeh.2010.00020 20514339 PMC2876873

[B221] Vaina L. M. Beardsley S. A. Rushton S. K. (2004). Optic Flow and Beyond. Dordrecht, Boston, London: Kluwer Academic Publishers

[B222] van Breugel F. Dickinson M. H. (2012). The visual control of landing and obstacle avoidance in the fruit fly *Drosophila melanogaster*. J. Exp. Biol. 215, 1783–1798 10.1242/jeb.066498 22573757

[B223] van Hateren J. H. (1992). Theoretical predictions of spatiotemporal receptive fields of fly LMCs, and experimental validation. J. Comp. Physiol. A 171, 157–170

[B224] van Hateren J. H. (1993). Three modes of spatiotemporal preprocessing by eyes. J. Comp. Physiol. A 172, 583–591 8331606 10.1007/BF00213681

[B225] van Hateren J. H. (1997). Processing of natural time series of intensities by the visual system of the blowfly. Vision Res. 37, 3407–3416 10.1016/S0042-6989(97)00105-3 9425553

[B226] van Hateren J. H. Kern R. Schwerdtfeger G. Egelhaaf M. (2005). Function and coding in the blowfly H1 neuron during naturalistic optic flow. J. Neurosci. 25, 4343–4352 10.1523/JNEUROSCI.0616-05.2005 15858060 PMC6725116

[B227] van Hateren J. H. Schilstra C. (1999). Blowfly flight and optic flow. II. Head movements during flight. J. Exp. Biol. 202, 1491–1500 10229695 10.1242/jeb.202.11.1491

[B228] Verspui R. Gray J. R. (2009). Visual stimuli induced by self-motion and object-motion modify odour-guided flight of male moths (*Manduca sexta* L.). Exp. Biol. 212, 3272–3282 10.1242/jeb.031591 19801432

[B229] Virsik R. Reichardt W. (1976). Detection and tracking of moving objects by the fly *Musca domestica*. Biol. Cybern. 23, 83–98

[B230] Wagner H. (1982). Flow-field variables trigger landing in flies. Nature 297, 147–148 2342582

[B231] Warzecha A.-K. Egelhaaf M. (1996). Intrinsic properties of biological motion detectors prevent the optomotor control system from getting unstable. Philos. Trans. R. Soc. B 351, 1579–1591

[B232] Warzecha A.-K. Egelhaaf M. (1997). How reliably does a neuron in the visual motion pathway of the fly encode behaviourally relevant information? Eur. J. Neurosci. 9, 1365–1374 9240394 10.1111/j.1460-9568.1997.tb01491.x

[B233] Warzecha A.-K. Egelhaaf M. (1999). Variability in spike trains during constant and dynamic stimulation. Science 283, 1927–1930 10.1126/science.283.5409.1927 10082467

[B234] Warzecha A.-K. Egelhaaf M. (2001). Neuronal encoding of visual motion in real-time, in Processing Visual Motion in the Real World: A Survey of Computational, Neural, and Ecological Constraints, eds Zanker J. M. Zeil J. (Berlin, Heidelberg, New York: Springer), 239–277

[B235] Warzecha A.-K. Egelhaaf M. Borst A. (1993). Neural circuit tuning fly visual interneurons to motion of small objects. I. Dissection of the circuit by pharmacological and photoinactivation techniques. J. Neurophysiol. 69, 329–339 8459270 10.1152/jn.1993.69.2.329

[B236] Warzecha A.-K. Horstmann W. Egelhaaf M. (1999). Temperature dependence of neuronal performance in the motion pathway of the blowfly *Calliphora erythrocephala*. J. Exp. Biol. 202, 3161–3170 10539965 10.1242/jeb.202.22.3161

[B237] Warzecha A.-K. Kretzberg J. Egelhaaf M. (1998). Temporal precision of the encoding of motion information by visual interneurons. Curr. Biol. 8, 359–368 10.1016/S0960-9822(98)70154-X 9545194

[B238] Warzecha A.-K. Kretzberg J. Egelhaaf M. (2000). Reliability of a fly motion-sensitive neuron depends on stimulus parameters. J. Neurosci. 20, 8886–8896 11102498 10.1523/JNEUROSCI.20-23-08886.2000 PMC6773076

[B239] Warzecha A.-K. Kurtz R. Egelhaaf M. (2003). Synaptic transfer of dynamical motion information between identified neurons in the visual system of the blowfly. Neuroscience 119, 1103–1112 10.1016/S0306-4522(03)00204-5 12831867

[B240] Wolf H. (2011). Odometry and insect navigation. J. Exp. Biol. 214, 1629–1641 10.1242/jeb.038570 21525309

[B241] Zeil J. (1986). The territorial flight of male houseflies (*Fannia canicularis*). Behav. Ecol. Sociobiol. 19, 213–219

[B242] Zeil J. (2012). Visual homing: an insect perspective. Curr. Opin. Neurobiol. 22, 285–293 10.1016/j.conb.2011.12.008 22221863

[B243] Zeil J. Boeddeker N. Stürzl W. (2009). Visual homing in insects and robots, in Flying Insects and Robots, eds Floreano D. Zufferey J. C. Srinivasan M. V. Ellington C. P. (Heidelberg, Dordtrecht, London, New York: Springer), 87–99

